# TREM2 Facilitates Myelin Debris Clearance but Exacerbates Chronic Inflammation and Fibrosis After Spinal Cord Injury

**DOI:** 10.1002/cns.70777

**Published:** 2026-02-09

**Authors:** Zhonghan Wu, Shuisheng Yu, Yixue Hu, Xuyang Hu, Linhan Yang, Yan Jiang, Mengdi Zhang, Yiyang Mu, Ziyu Li, Fei Yao, Dasheng Tian, Juehua Jing, Li Cheng

**Affiliations:** ^1^ Department of Orthopaedics The Second Affiliated Hospital of Anhui Medical University Hefei China; ^2^ Institute of Orthopaedics, Research Center for Translational Medicine The Second Affiliated Hospital of Anhui Medical University Hefei China; ^3^ Department of Rehabilitation Medicine The Second Affiliated Hospital of Anhui Medical University Hefei China

**Keywords:** disease‐associated microglia, foamy macrophage, myelin debris, spinal cord injury, TREM2

## Abstract

**Background:**

The accumulation of myelin debris after spinal cord injury (SCI) inhibits axon regeneration and remyelination. Triggering receptor expressed on myeloid cell 2 (TREM2) is crucial for cellular debris clearance and disease‐associated microglia (DAM) activation. However, whether TREM2 mediates these processes after SCI remains unclear.

**Methods:**

A mouse model of spinal cord crush injury was employed. Female *TREM2* ^−/−^ mice were used to delete TREM2, while COG1410 was administered to activate TREM2 in female wild‐type mice. Tissue immunostaining and western blotting were performed to analyze TREM2 expression after SCI. Tissue immunostaining was conducted to evaluate the cellular origin of TREM2 and its impact on phagocytosis, foamy macrophage formation, DAM activation, axon regeneration, and neuronal survival. Basso Mouse Scale and footprint analysis were used to evaluate locomotor function recovery.

**Results:**

TREM2 was primarily localized to Iba1^+^ macrophages/microglia around the lesion core, with its expression increasing during the subacute stage, peaking at 7 days post‐injury. TREM2 deficiency impaired engulfment and degradation of myelin debris, increased foamy macrophage formation, and hindered DAM activation. In vivo rescue experiments further confirmed that TREM2 promotes DAM activation via the PI3K/AKT pathway. However, TREM2 exacerbated fibrosis, as indicated by increased extracellular matrix deposition, enhanced fibroblast accumulation, and widespread inflammation. COG1410‐mediated long‐term activation of TREM2 impaired long‐term locomotor function recovery, inhibited axon regeneration, and reduced neuronal survival, whereas short‐term activation improved early locomotor function without structural neuroprotection.

**Conclusions:**

Our study suggests that TREM2 promotes myelin debris clearance but exacerbates chronic inflammation and fibrosis after SCI. These findings underscore the promise of TREM2 as a target for developing effective treatment strategies for SCI.

## Graphical abstract

After spinal cord injury (SCI), TREM2 promotes myelin debris clearance and disease‐associated microglia (DAM) activation that support early repair. However, sustained TREM2‐dependent DAM activation may aggravate chronic inflammation and fibrosis, whereas TREM2 deficiency alleviates these effects, identifying TREM2 as a key regulator of microglial responses following SCI.

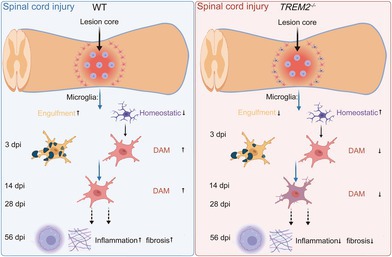

**Abbreviations**
AD: Alzheimer's disease
BMS: Basso Mouse Scale
CNS: central nervous system
COG1410: synthetic peptide mimicking TREM2 ligand
CSPG: chondroitin sulfate proteoglycan
DAM: disease‐associated microglia
dMBP: denatured myelin basic protein
DMSO: dimethyl sulfoxide
dpi: days post‐injury
DSA: Donkey Serum Albumin
ECM: extracellular matrix
EES: epidural electrical stimulation
FC: fold change
FDR: false discovery rate
GFAP: glial fibrillary acidic protein
IS: ischemic stroke
LR: lactated Ringer's solution
MBP: myelin basic protein
ORO: Oil Red O
PBS: phosphate‐buffered saline
PFA: paraformaldehyde
PI3K/AKT: phosphatidylinositol 3‐kinase/protein kinase B
SCI: spinal cord injury
TBI: traumatic brain injury
TREM2: triggering receptor expressed on myeloid cell 2
UMIs: unique molecular identifiers
WT: wild‐type

## 1 Introduction

Spinal cord injury (SCI) is a devastating disease that irreversibly damages neural tissues, leading to permanent motor, sensory, and autonomic dysfunction, with no curative treatment currently available, severely impacting the quality of life for millions of people worldwide [1, 2]. After SCI, the accumulation of myelin debris activates macrophages/microglia, provoking complicated inflammatory responses, which can exacerbate neuronal damage and cell death [1, 2]. Myelin debris also hinders remyelination to impair the repair process after injury [3]. Therefore, effective clearance of myelin debris is crucial for the management of SCI [4, 5]. Microglia play an essential role in the initial response to SCI by efficiently processing myelin debris [4, 5, 6]. Disease‐associated microglia (DAM) in SCI originate from baseline microglia [7], yet the current understanding of their roles in SCI pathology remains limited [8]. Peripheral macrophages become predominantly involved in phagocytosis at later stages, but they are less effective at the degradation of myelin debris [4, 6]. However, the key molecule that mediates the engulfment of myelin debris by macrophages/microglia after SCI is unclear.

Triggering receptor expressed on myeloid cell 2 (TREM2) is an immunoglobulin receptor expressed specifically by CNS macrophages/microglia, serving as a key signaling molecule for lipid ligand recognition, phagocytosis, and lipid metabolism [9, 10]. Upon activation, TREM2 initiates downstream signaling pathways that promote phagocytosis, cell proliferation, DAM activation, as well as glucose and lipid metabolism [11, 12]. A previous study has shown the distinct phenotypes of macrophages/microglia dependent on TREM2 levels at the acute stage of SCI [13], whereas the expression pattern of TREM2 at the subacute and intermediate stages [14] of SCI is unknown. In addition, whether TREM2 deficiency affects the clearance of myelin debris and DAM activation after SCI remains unclear.

Our study revealed that TREM2 was predominantly expressed on macrophages/microglia after SCI, with its levels peaking in the subacute phase and declining later. Functionally, TREM2 deficiency impaired myelin debris clearance and promoted foamy macrophage accumulation; TREM2 deficiency also suppressed DAM activation, which may have contributed to reduced fibrosis. Mechanistically, TREM2‐deficient DAM may fail to activate key microglial activation and inflammation‐related pathways, including the phosphatidylinositol 3‐kinase/protein kinase B (PI3K/AKT) signaling pathway. Notably, systemic administration of the AKT activator SC79 partially restored DAM activation. Long‐term TREM2 activation with COG1410 impaired functional recovery, axonal regeneration, and neuronal survival, whereas short‐term activation produced an early functional benefit without contributing to axonal regeneration or neuronal preservation. Together, these results reveal the complex involvement of TREM2 in microglial response after SCI and suggest that targeting TREM2 may offer pharmacological or gene therapy strategies for SCI.

## 2 Materials and Methods

### 2.1 Animals and Spinal Cord Crush Injury Model

The Animal Ethics Committee of Anhui Medical University formally approved all animal experiments (approval number: LLSC20232105). Female wild‐type (WT) mice, ranging in age from 8 to 10 weeks, were procured from the Animal Experiment Center at Anhui Medical University. In addition, *TREM2* ^−/−^ mice (NM‐KO‐190402) of the same age were acquired from Shanghai Model Organisms (Shanghai, China), while *CCR2* ^RFP^ *CX3CR1* ^GFP^ dual‐reporter mice (JAX:032127) of the same age were supplied by The Jackson Laboratory (Bar Harbor, USA). Separately, *CX3CR1* ^GFP^ single‐reporter mice (JAX: 005582) were obtained from The Jackson Laboratory (Bar Harbor, USA). Furthermore, the *TREM2* ^−/−^; *CX3CR1* ^GFP^ mice included in this study were generated by crossing *TREM2* ^−/−^ mice with *CX3CR1* ^GFP^ mice. All offspring were genotyped by PCR following the provider's protocol, and mice carrying the expected genotype were selected for experiments (detailed in Supplementary Methods (Data S1)). All mice used in this study were of specific‐pathogen‐free (SPF) grade and maintained at a weight of 20–25 g. The care of the animals was carried out in accordance with the established ethical guidelines for animal experimentation.

The establishment of the spinal cord crush model was executed as previously detailed [4]. Mice were anesthetized with a combination of zoletil (40 mg/kg) and xylazine hydrochloride (5–10 mg/kg). The T10 spinal cord was then exposed, and #5 dummy tweezers were inserted vertically into the gap between the spinal cord and the spinal canal bilaterally. The spinal cord crush was maintained for 5 s before wound closure. Postoperatively, mice were administered anti‐infective treatment and received twice‐daily assistance with urination.

### 2.2 Therapeutic Agents and Routes of Administration

The therapeutic agent, COG1410, is a synthetic peptide that mimics the natural ligand of TREM2, activates the receptor [15, 16]. COG1410 (HY‐P2136, MedChemExpress, Monmouth Junction, NJ, USA) was prepared using lactated Ringer's solution (LR) from Baxter Healthcare Corporation (Deerfield, IL, USA). The concentration of the solution was 200 μg/mL, and the dosage was 0.6 mg/kg body weight. The initial dose was administered intravenously (I.V.) via the tail vein within 5 min post‐injury, and subsequent treatments were delivered once daily via intraperitoneal injection (I.P.), beginning 3 h after injury. COG1410 (Ac‐Ala‐Ser‐Aib‐Leu‐Arg‐Lys‐Leu‐Aib‐Lys‐Arg‐Leu‐Leu‐NH2) was used as a functional apoE‐mimetic peptide. In parallel, a non‐functional scrambled peptide (Ac‐Leu‐Arg‐Aib‐Ala‐Lys‐Leu‐Ser‐Aib‐Lys‐Arg‐Leu‐Leu‐NH2) with the same amino acid composition was designed as a control to maintain similar physicochemical properties while avoiding bioactive motifs. The scrambled peptide was custom‐synthesized by TargetMol (Shanghai, China) using standard Fmoc chemistries [17] on a Symphony X peptide synthesizer (Gyros Protein Technologies, Tucson, AZ, USA). After synthesis, the peptide was purified by high‐performance liquid chromatography (HPLC, purity ≥ 95%), verified by mass spectrometry, and tested for endotoxin levels (LAL), which were < 0.01 EU/μg. Control animals were treated with either an equivalent volume of LR or a scrambled peptide matched in dosing schedule and route.

SC79, the therapeutic agent used in the in vivo rescue experiment, is a highly specific and blood–brain barrier (BBB)‐permeable AKT activator that activates cytosolic AKT in models of Alzheimer's disease (AD), ischemic stroke (IS), and SCI [18, 19, 20]. For in vivo use, SC79 (HY‐18749, MedChemExpress, Monmouth Junction, NJ, USA) was dissolved in 5% dimethyl sulfoxide (DMSO; HY‐Y0320C, MedChemExpress, Monmouth Junction, NJ, USA)/saline to a final concentration of 5 mg/mL. Mice received SC79 at a dosage of 10 mg/kg body weight via intraperitoneal injection. SC79 was administered to WT and *TREM2* ^−/−^ mice 0.5 h prior to SCI, followed by once‐daily injections post‐injury to induce AKT phosphorylation in microglia. The control group received an equivalent volume of 5% DMSO/saline.

### 2.3 Tissue Preparation and Immunostaining

Following transcardial perfusion with 40 mL of 0.1 M phosphate‐buffered saline (PBS; Biosharp, Hefei, China) to clear blood, 20 mL of 4% paraformaldehyde (PFA; Biosharp, Hefei, China) was used for fixation. A 1‐cm spinal cord segment, centered on the core of the T10 lesion, was immersed in 4% PFA for 5–6 h before being dehydrated in a 30% sucrose solution overnight at 4°C. Tissues were sectioned at a thickness of 16 μm using a frozen microtome (NX50, Thermo Fisher Scientific, Waltham, MA, USA).

The sections were then permeabilized with 0.3% Triton X‐100 and blocked with 10% donkey serum albumin (DSA) for 1 h at room temperature. After blocking, primary antibodies were applied overnight at 4°C in the humidity chamber. The antibodies used in this study included the following: TREM2 (10 μg/mL, AF1729, R&D Systems, USA), PDGFRβ (5 μg/mL, AF1042‐SP, R&D Systems, USA), CD31 (1:100, AF3628, R&D Systems, USA), 5‐hydroxytryptamine (5‐HT) (1:5000, 20,079, Immunostar, Hudson, WI, USA), Iba1 (1:100, NB100‐1028, Novus, St Louis, MO, USA), CCR2 (1:100, NBP1‐48338, Novus, St Louis, MO, USA), fibronectin (1:100, 15613‐1‐AP, Proteintech, China), laminin (1:100, 23498‐1‐AP, Proteintech, China), glial fibrillary acidic protein (GFAP) (1:400, 13‐0300, Thermo Fisher Scientific, Waltham, MA, USA), CD68 (1:400, MCA1957, Bio‐Rad, Hercules, CA, USA), CSPG (1:200, C8035, Sigma‐Aldrich, USA), collagen I (1:200, ab21286, Abcam, USA), CST7 (1:100, PA5‐103772, Thermo Fisher Scientific, Waltham, MA, USA), IGF1 (1:100, ab223567, Abcam, USA), P2ry12 (1:500, EPR26298‐93, Abcam, USA), NeuN (1:100, EPR12763, Abcam, USA), AKT (1:100, MA5‐41139, Thermo Fisher Scientific, Waltham, MA, USA), GSK3β (25 μg/mL, MAB2506, R&D Systems, USA), p‐AKT (1:100, sc‐514,032, Santa Cruz, USA), and p‐GSK3β (1:100, MA5‐14873, Thermo Fisher Scientific, Waltham, MA, USA).

The sections were subsequently incubated with fluorescent secondary antibodies for 1 h at room temperature, protected from light. Sections were coverslipped with an anti‐fade reagent containing DAPI (P0131, Beyotime, China) after washing with PBS.

### 2.4 Evaluation of Neurological Recovery in Mice

Locomotor function recovery was assessed using the Basso Mouse Scale (BMS) and footprint analysis. BMS scores [21, 22], a gold standard for evaluating hind limb function after SCI, were recorded pre‐operation and at 3, 7, 14, 28, and 56 days post‐injury (dpi), focusing on hind ankle movement and limb coordination. Two independent evaluators scored each mouse, and the average score was calculated. For the footprint analysis [23], mice were allowed to walk on A4 paper. Their front paws were dipped in green ink, and their hind paws in red ink. Hind limb function was assessed using step length, step width, and rotation angle.

### 2.5 Image Acquisition and Quantitative Analysis

Immunostaining images were acquired using a Zeiss Axio Scope A1 fluorescence microscope and a Zeiss LSM880 laser scanning confocal microscope. Quantitative analysis of the images was performed using ImageJ version 2.0 (NIH, United States) and GraphPad Prism version 10.0 (NIH, United States) software. All analyses were conducted in a blinded fashion.

To examine the cellular origin of TREM2 after SCI, three sections per sample were labeled with TREM2, Iba1 (macrophages/microglia), CD31 (endothelial cells), PDGFRβ (fibroblasts), GFAP (astrocytes), CX3CR1‐GFP (microglia), and CCR2‐RFP (macrophages). For each section, three independent 40× images were randomly acquired, and the mean of these images was employed as the final data point for each specimen. The data were presented as the percentage of TREM2^+^Iba1^+^, TREM2^+^CD31^+^, TREM2^+^PDGFRβ^+^, TREM2^+^GFAP^+^, TREM2^+^CX3CR1‐GFP^+^CCR2‐RFP^−^, and TREM2^+^CCR2‐RFP^+^ cells normalized to the overall number of TREM2^+^ cells.

To assess the expression level of TREM2 following SCI, three sections per sample were labeled with TREM2 and GFAP (astrocytes). For each section, three independent 40× images were randomly acquired, and the average intensity was employed as the final data point for each specimen. The results were presented as TREM2 mean fluorescence intensity.

To investigate DAM generation and microglial homeostasis, CST7 and IGF1 were stained as DAM markers, and the data were presented as the number of Iba1^+^CST7^+^ or Iba1^+^IGF1^+^ cells per mm^2^. P2ry12 was stained as a microglial homeostasis marker, and the data were presented as the number of P2ry12^+^ cells per mm^2^. To evaluate PI3K/AKT pathway activation, triple immunostaining for p‐AKT/AKT/Iba1, as well as for p‐GSK3β/GSK3β/Iba1, was performed. Pathway activity was primarily quantified as the ratio of mean fluorescence intensity of p‐AKT to AKT or p‐GSK3β to GSK3β within Iba1^+^ cells.

To trace the pathological characteristics of lesion sites, GFAP, Fibronectin, PDGFRβ, CSPG, collagen I, and laminin were used for immunostaining. Immunostaining intensity at the lesion site (a 100‐μm wide region at the lesion core) was measured using ImageJ software and normalized to the intensity in the intact (proximal) region of the spinal cord.

Phagocytosis‐related images were processed and analyzed using Imaris Software (Bitplane, Switzerland) (detailed in Supplementary Methods (Data S1)). Lipid metabolism dysfunction was indicated by the presence of foam cells. Sections were stained with Oil Red O (G1263, Solarbio, China) to identify lipid‐laden macrophages, known as foamy macrophages [24]. The pixel area of the ORO^+^ positive region was extracted, and the data were presented as a ratio of the ORO^+^ positive area to the entire view area. To further characterize lipid metabolism dysfunction in specific myeloid populations, tissues were stained with BODIPY (C2053S, Beyotime, China) together with markers distinguishing microglia (CX3CR1‐GFP^+^CCR2^−^) and infiltrating macrophages (CCR2^+^) [3]. The data were quantified as the number of BODIPY^+^CX3CR1‐GFP^+^CCR2^−^ microglia or BODIPY^+^CCR2^+^ macrophages per mm^2^.

Neuronal survival was assessed by categorizing the sections into three regions based on their proximity to the lesion core: Z1 (0–250 μm), Z2 (250–500 μm), and Z3 (1000–1250 μm), as previously described [25]. The sections were labeled with NeuN and GFAP. The data were presented as the density of NeuN^+^ cells. For the quantification of the regenerated descending serotonergic (5‐HT^+^) axons, the sections were labeled with 5‐HT and GFAP. The 5‐HT immunoreactivity was normalized to the GFAP^−^ area at low magnification, and the mean value was employed as the final data point for each specimen.

### 2.6 Statistical Analysis

Data are presented as means ± standard error of the mean (SEM), with at least three biological replicates per group, and individual data points are shown in the figures. Statistical comparisons between two groups were performed using Student's *t*‐test, and one‐way/two‐way analysis of variance (ANOVA) followed by Tukey's post hoc test was applied for multiple groups. Data analysis and graphing were performed using GraphPad Prism 10.0 (GraphPad, United States), and statistical significance was determined with a significance threshold of *p* < 0.05.

### 2.7 RNA‐Seq Data Analysis

Public single‐cell RNA‐seq data (GSE198852) were analyzed using the NovelBrain Cloud Platform. Differentially expressed genes (DEGs) were filtered using DESeq2 algorithm (FC > 1.5, FDR < 0.05), and pathway enrichment was performed via Kyoto Encyclopedia of Genes and Genomes (KEGG) and Gene Ontology (GO) analysis (detailed in Supplementary Methods (Data S1)).

## 3 Results

### 3.1 TREM2 is Primarily Expressed on Macrophages/Microglia After SCI

**FIGURE 1:**
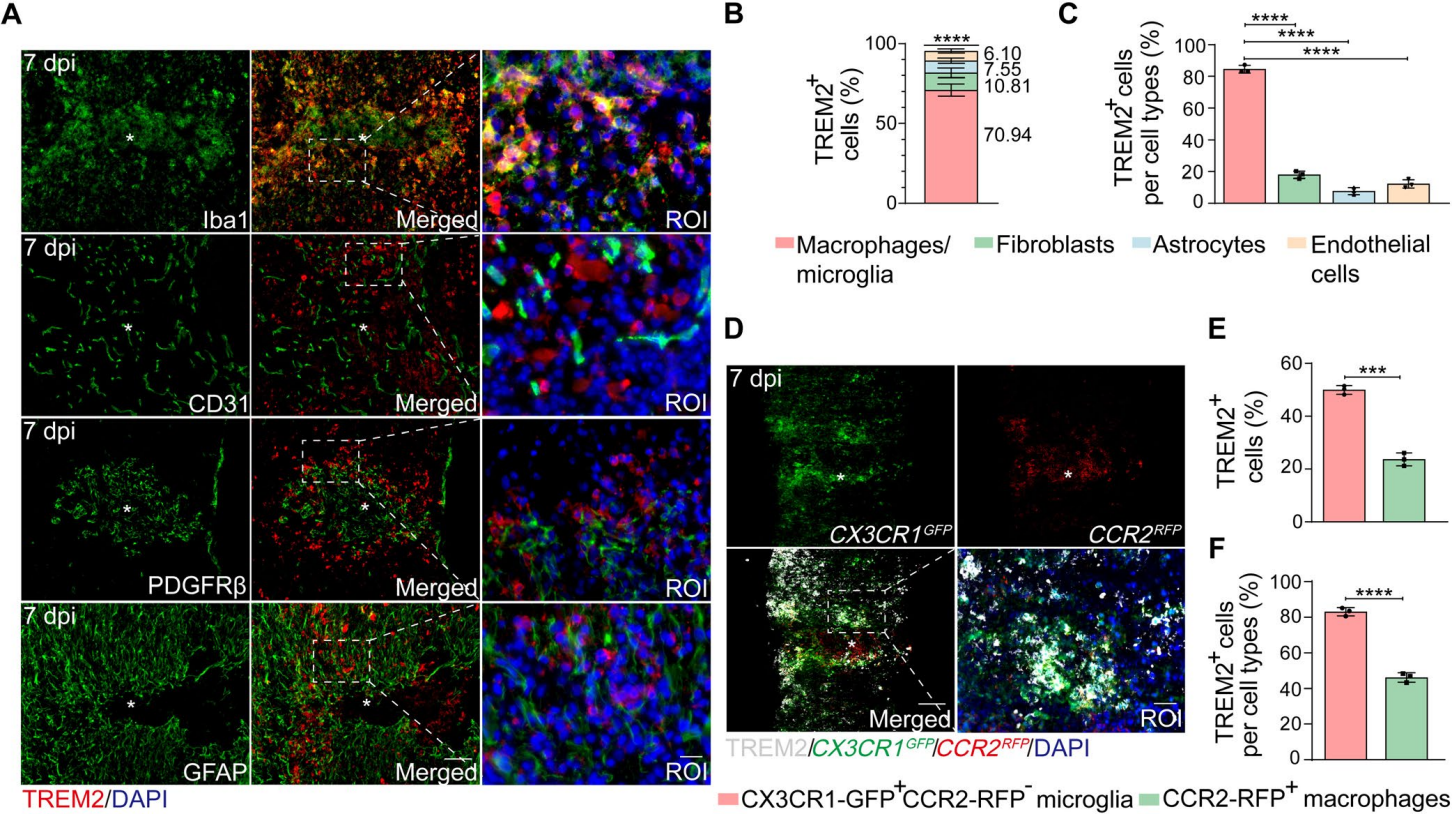
TREM2 is primarily expressed on macrophages/microglia after SCI. (A) Representative immunostaining image of Iba1 (green), CD31 (green), PDGFRβ (green), or GFAP (green), TREM2 (red), and DAPI (blue) in sagittal sections at 7 dpi. Scale bars: Low magnification, 100 μm; high magnification, 20 μm. The asterisk indicates the lesion core. (B) Bar graphs show the percentage of each cell type expressing TREM2 from the mice described in (A). Asterisks show a statistically significant difference between macrophages/microglia and all other TREM2‐expressing cells. (C) Bar graphs show the percentage of TREM2‐expressing cells within each cell type from the mice described in (A). Asterisks show statistically significant differences in macrophages/microglia and all other TREM2‐expressing cells. (D) Representative immunostaining images of CX3CR1‐GFP (green), CCR2‐RFP (red), and TREM2 (white) in sagittal sections at 7 dpi. Scale bars: Low magnification, 100 μm; high magnification, 20 μm. The asterisk indicates the lesion core. (E) Bar graphs show the percentage of microglia and macrophages expressing TREM2 from the mice described in (D). Asterisks show a statistically significant difference between microglia and macrophages. (F) Bar graphs show the percentage of TREM2‐expressing cells within microglia and macrophages from the mice described in (D). Asterisks show statistically significant differences in microglia and macrophages. Statistical significance between experimental groups was calculated by one‐way ANOVA followed by Tukey's post hoc test (B, C), unpaired Student's *t*‐test (E, F). ***p < 0.001, ****p < 0.0001. Data are presented as mean ± SEM; each point represents an individual mouse.

To determine the cellular origin of TREM2 after SCI, we evaluated its co‐localization with key immune and structural cells at the lesion site, such as macrophages/microglia (Iba1^+^), vascular endothelial cells (CD31^+^), astrocytes (GFAP^+^), and fibroblasts (PDGFRβ^+^). The initial one‐week post‐injury represents a highly dynamic phase, offering a critical therapeutic window for targeting multiple pathological processes. Thus, we chose to examine spinal cord tissue at 7 dpi. Immunostaining revealed a high degree of co‐localization between TREM2 and Iba1^+^ macrophages/microglia at 7 dpi, with TREM2^+^Iba1^+^ cells accounting for 70.9% of TREM2^+^ cells and 84.5% of Iba1^+^ cells (Figure 1A–C). In contrast, minimal co‐localization was observed between TREM2 and PDGFRβ^+^ fibroblasts, CD31^+^ vascular endothelial cells, or GFAP^+^ astrocytes (Figure 1A–C). Considering that Iba1 cannot differentiate microglia from macrophages after injury, we further examined TREM2 expression in injured tissues of *CCR2* ^RFP^ *CX3CR1* ^GFP^ dual‐reporter mice at 7 dpi. The results showed that TREM2 was primarily expressed by CX3CR1‐GFP^+^CCR2‐RFP^−^ microglia outside the lesion core, with partial expression by CCR2‐RFP^+^ macrophages accumulated at the lesion core (Figure 1D–F). These results indicate that TREM2 is primarily expressed on macrophages/microglia, particularly on activated microglia.

### 3.2 TREM2 Expression Is Upregulated After SCI

**FIGURE 2:**
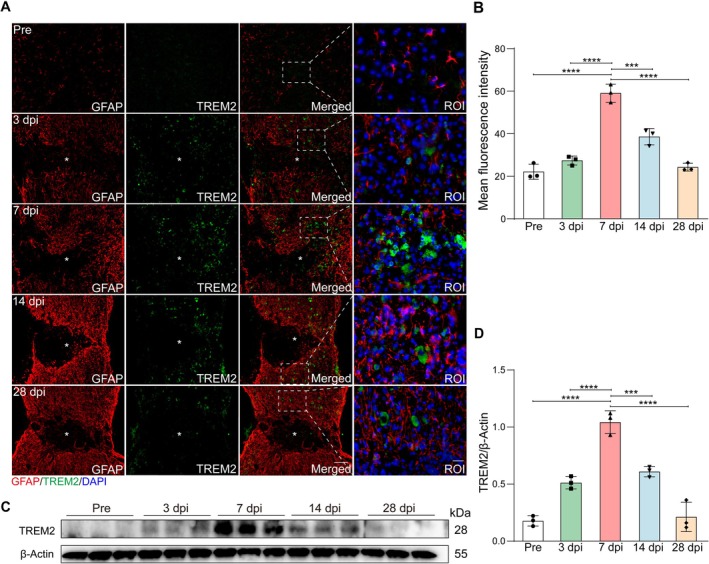
Spatiotemporal expression and upregulation of TREM2 after SCI. (A) Double immunostaining labeling of spinal cord sagittal sections shows the spatiotemporal distribution of GFAP (red) and TREM2 (green) pre‐operation and at 3, 7, 14, and 28 dpi. Scale bar: Low magnification, 100 μm; high magnification, 20 μm. (B) Quantification of the TREM2 expression in (A). (C) Western blotting analysis shows significant upregulation of TREM2 at 7 dpi compared to Pre. (D) Quantification of TREM2 expression in (C). Statistical significance between experimental groups was calculated by one‐way ANOVA followed by Tukey's post hoc test (B, D). ***p < 0.001, ****p < 0.0001. Data are presented as mean ± SEM; each point represents an individual mouse.

To detect changes in TREM2 expression at the subacute (2–14 days) and intermediate (14 days to 6 months) stages of SCI, we extracted spinal cord tissue pre‐operation and at 3, 7, 14, and 28 dpi. We analyzed the spatiotemporal expression of TREM2 following SCI through immunostaining and western blotting. Macrophages/microglia exhibit the critical window of activity at the subacute stage, during which they acquire diverse transcriptional profiles to engage in phagocytosis, lipid metabolism, and extracellular matrix (ECM) organization [26]. The immunostaining results showed that TREM2^+^ cells were mainly distributed outside the lesion core, with partial localization to the core from 3 to 28 dpi (Figure 2A). Compared to pre‐operation levels, TREM2 mean fluorescence intensity increased during the subacute phase (peaking at 7 dpi), and subsequently declined during the intermediate stage of SCI (Figure 2A,B), corresponding to the spatial and temporal pattern of macrophages/microglia activity. The western blotting analysis further revealed that TREM2 expression was significantly increased at 7 dpi compared to pre‐operation levels (Figure 2C,D). Notably, the high expression period of TREM2 is consistent with the active phagocytosis phase of macrophages/microglia after SCI [6], indicating the potential association between TREM2 and phagocytosis of macrophages/microglia after SCI.

### 3.3 TREM2 Deficiency Impairs Myelin Debris Clearance

**FIGURE 3:**
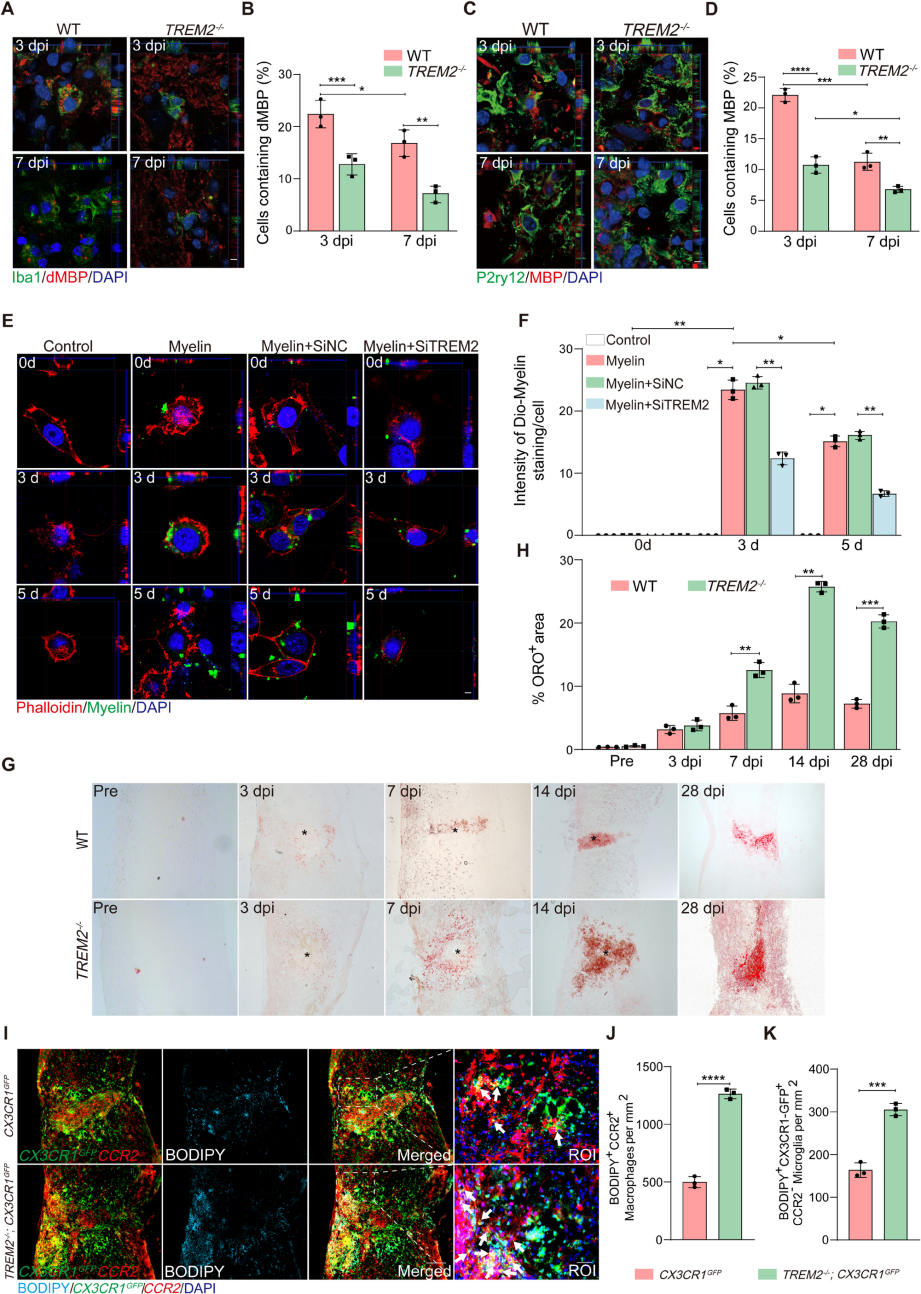
TREM2 deficiency in macrophages/microglia impairs myelin debris clearance. (A, C) Representative confocal z‐stack and corresponding 3D surface rendering show volume reconstruction of (A) microglia (green) and dMBP (red), and (C) microglia (green) and MBP (red), detected at 3 and 7 dpi in the WT and *TREM2* ^−/−^ mice. Scale bars: 10 μm. (B, D) Quantification of engulfed dMBP or MBP within cells shown in (A, C). (E) Representative immunofluorescence images of primary microglia (phalloidin staining, red) incubated with Dio‐myelin (1 mg/mL, green) for 0–5 days in vitro. (F) Quantification of intensity of Dio‐Myelin staining/cell in (E). (G) Representative immunostaining images of Oil Red O staining (ORO) pre‐operation and at 3, 7, 14, and 28 dpi in WT and *TREM2* ^−/−^ mice. ORO is used to assess foamy macrophages. Scale bars, 200 μm. (H) Quantification of the ORO staining area in (G). (I) Macrophages/microglia in *TREM2* ^−/−^; *CX3CR1* ^GFP^ mice show greater accumulation of lipid droplets (BODIPY^+^) than those in *CX3CR1* ^GFP^ mice at 14 dpi. (J, K) Quantification of BODIPY^+^ macrophages and microglia numbers in *CX3CR1* ^GFP^ and *TREM2* ^−/−^; *CX3CR1* ^GFP^ mice at 14 dpi. Statistical significance between experimental groups was calculated by two‐way ANOVA followed by Tukey's post hoc test (B, D, F, H), unpaired Student's *t*‐test (J), and (K). *p < 0.05, **p < 0.01, ***p < 0.001, ****p < 0.0001. Data are presented as mean ± SEM; each point represents an individual mouse.

To assess the contribution of TREM2 to myelin debris clearance, we first quantified the phagocytosed myelin or phagocytosed myelin debris within microglia after SCI. The intact myelin component was labeled with an anti‐MBP antibody [27], and myelin debris was labeled with an anti‐dMBP antibody that selectively recognizes MBP epitopes exposed following myelin degradation [24, 28]. Three‐dimensional confocal imaging and phagocytic evaluation showed an evident decrease in dMBP (red) content within Iba1^+^ microglia (green) at 7 dpi compared to 3 dpi in WT mice (Figure 3A,B), which is consistent with that microglia play a major role in phagocytosis at 3 dpi [6, 29]. Additionally, a significant reduction in dMBP content within cells per field was observed at the same time point in *TREM2* ^−/−^ mice compared to WT mice (Figure 3A,B). Similar outcomes were observed in the analysis of MBP (red) engulfment by P2ry12^+^ microglia (green) (Figure 3C,D). To further validate these findings, we established a primary microglia culture system for in vitro experiments. First, microglia were transfected with a siRNA targeting TREM2 (SiTREM2) or a negative control siRNA (SiNC) to modulate TREM2 expression. Subsequently, 24 h after siRNA transfection, myelin debris was added to a subset of untransfected microglia (Myelin group), the SiNC‐transfected microglia (Myelin+SiNC group), and the SiTREM2‐transfected microglia (Myelin+SiTREM2 group). Western blotting was performed after the treatment. The results showed that TREM2 expression was significantly upregulated in the Myelin group relative to the untreated control group (Figure S1A,B). As expected, the Myelin+SiTREM2 group exhibited a substantial reduction in TREM2 expression relative to the Myelin+SiNC group (Figure S1A,B). In addition, the co‐localization of DiO‐myelin with microglia revealed that microglial phagocytic activity increased markedly at 3 days after induction, reaching a peak, and subsequently declined by 5 days (Figure 3E,F). These findings further support our in vivo observations, indicating that microglia exert robust phagocytic activity primarily during the acute phase. Notably, at both 3 days and 5 days, microglia in the Myelin+SiTREM2 group exhibited substantially reduced internalization of DiO‐myelin compared with those in the Myelin+SiNC group (Figure 3E,F). Collectively, these results strongly suggest that TREM2 deficiency can impair myelin debris engulfment by microglia.

Excessive myelin debris is delivered to lysosomes for degradation after being engulfed by macrophages and is eventually converted into intracellular lipid droplets containing neutral lipids, leading to foamy macrophage formation [3, 24]. A previous study has shown that TREM2‐deficient microglia are able to engulf some myelin debris but are deficient in clearing myelin cholesterol, leading to lipid accumulation [30]. Next, we sought to investigate whether lipid accumulation exists in TREM2‐deficient macrophages/microglia after SCI. Oil Red O staining is widely utilized to specifically detect intracellular neutral lipids [24, 29]. Additionally, BODIPY, a fluorescent dye that specifically detects lipid droplets, was chosen to jointly evaluate lipid homeostasis and lipid accumulation in macrophages/microglia [3, 31]. As shown in Figure 3G,H, the *TREM2* ^−/−^ mice exhibited a significantly larger area of Oil Red O staining at the lesion core compared to WT mice at 7, 14, and 28 dpi, suggesting an increased number of foamy macrophages in the *TREM2* ^−/−^ mice. Additionally, as shown in Figure 3I–K, significantly increased BODIPY^+^CCR2^+^ macrophages and more BODIPY^+^CX3CR1‐GFP^+^CCR2^−^ microglia at the lesion site were observed in *TREM2* ^−/−^; *CX3CR1* ^GFP^ mice compared to *CX3CR1* ^GFP^ mice at 14 dpi, indicating an increase in lipid‐laden macrophages/microglia in *TREM2* ^−/−^; *CX3CR1* ^GFP^ mice. These results suggest that TREM2 deficiency disrupts lipid homeostasis of macrophages/microglia and promotes foamy macrophage formation after SCI.

### 3.4 TREM2 Deficiency May Dampen the PI3K/AKT Pathway, Impair DAM Activation, and Maintain Microglia in a Homeostatic State

**FIGURE 4:**
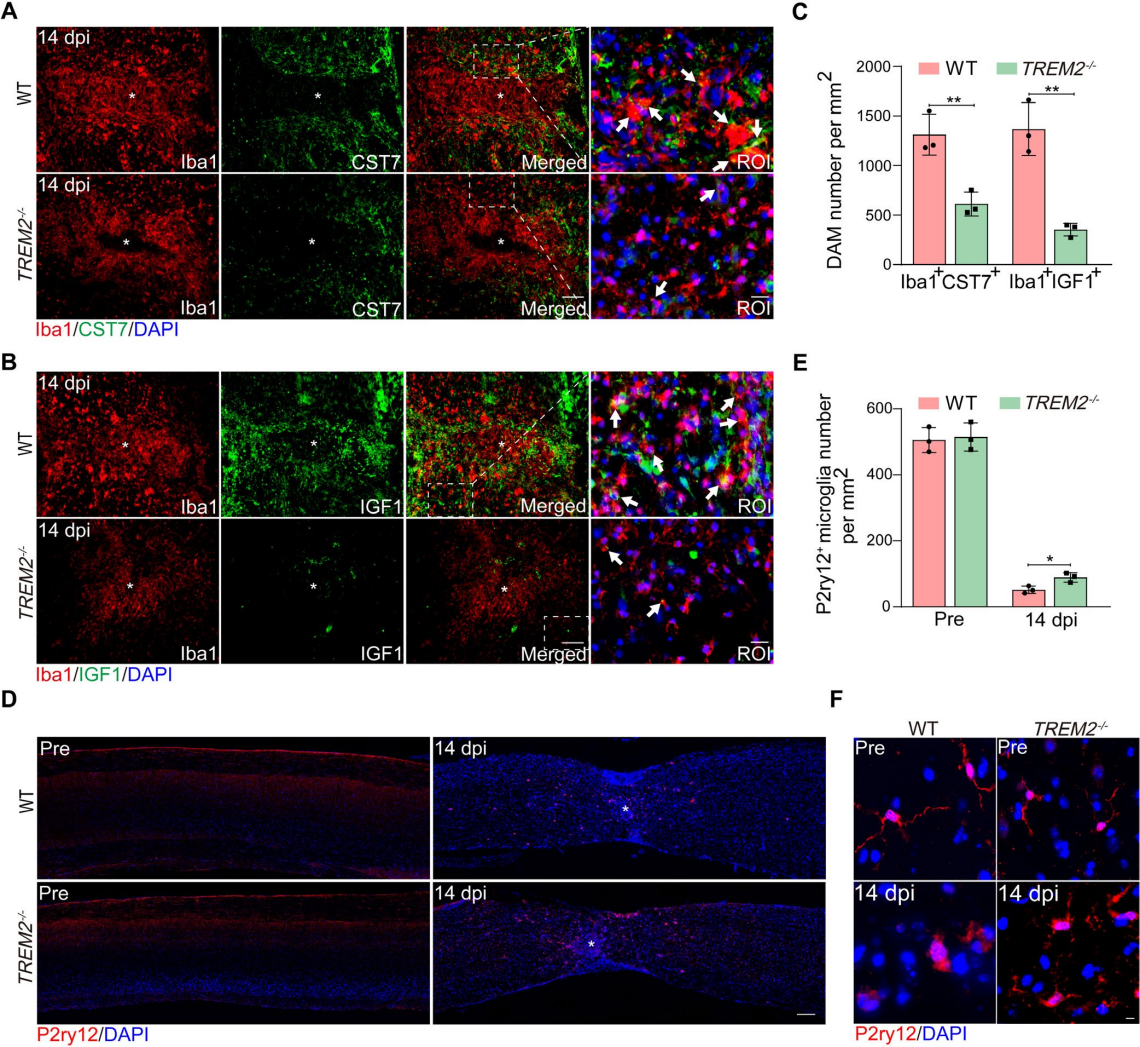
TREM2 deficiency abolishes CST7^+^ and IGF1^+^ DAM after SCI. (A, B) Microglia show lower expression of CST7 and IGF1 in *TREM2* ^−/−^ mice than in WT mice at 14 dpi. Scale bars: Low magnification, 100 μm; high magnification, 20 μm. (C) Quantification of Iba1^+^CST7^+^ and Iba1^+^IGF1^+^ DAM number in WT and *TREM2* ^−/−^ mice at 14 dpi. (D) Representative images of P2RY12^+^ microglia immunostaining in WT and *TREM2* ^−/−^ mice pre‐operation and at 14 dpi. Scale bars: 200 μm. (E) Quantification of P2RY12^+^ microglia number in WT and *TREM2* ^−/−^ mice in (D). (F) Representative images of P2RY12^+^ microglia immunostaining in WT and *TREM2* ^−/−^ mice pre‐operation and at 14 dpi. Scale bars: 10 μm. Statistical significance between experimental groups was calculated by unpaired Student's *t*‐test (C), two‐way ANOVA followed by Tukey's post hoc test (E). *p < 0.05, **p < 0.01. Data are presented as mean ± SEM; each point represents an individual mouse.

A recent study has shown that homeostatic microglia are typically activated into DAM after SCI, a process that occurs in two stages: Stage 1 and Stage 2 [7]. These cells can be induced by myelin debris and may participate in various processes [32]. Given that TREM2 promotes the expression of DAM markers after traumatic brain injury (TBI) [33], we hypothesized that TREM2 may also induce the DAM activation after SCI. Compared to WT mice, significantly fewer stage 2 DAM (Iba1^+^CST7^+^ or Iba1^+^IGF1^+^) were observed in *TREM2* ^−/−^ mice at 14 dpi (Figure 4A–C). Similar results were found at 3 dpi and 28 dpi (Figure S2A–F). During the complete DAM activation process, the expression of P2ry12, a homeostatic marker of microglia, typically decreases in stage 1 [7]. However, *TREM2* ^−/−^ mice exhibited a greater number of P2ry12^+^ microglia than WT mice at 14 dpi (Figure 4D,E). These microglia exhibited an intermediate morphology, displaying some characteristics of both highly ramified and amoeboid forms (Figure 4F), further indicating that TREM2‐deficient microglia were not completely activated. Therefore, TREM2 is crucial for the complete activation of microglia to DAM after SCI.

**FIGURE 5:**
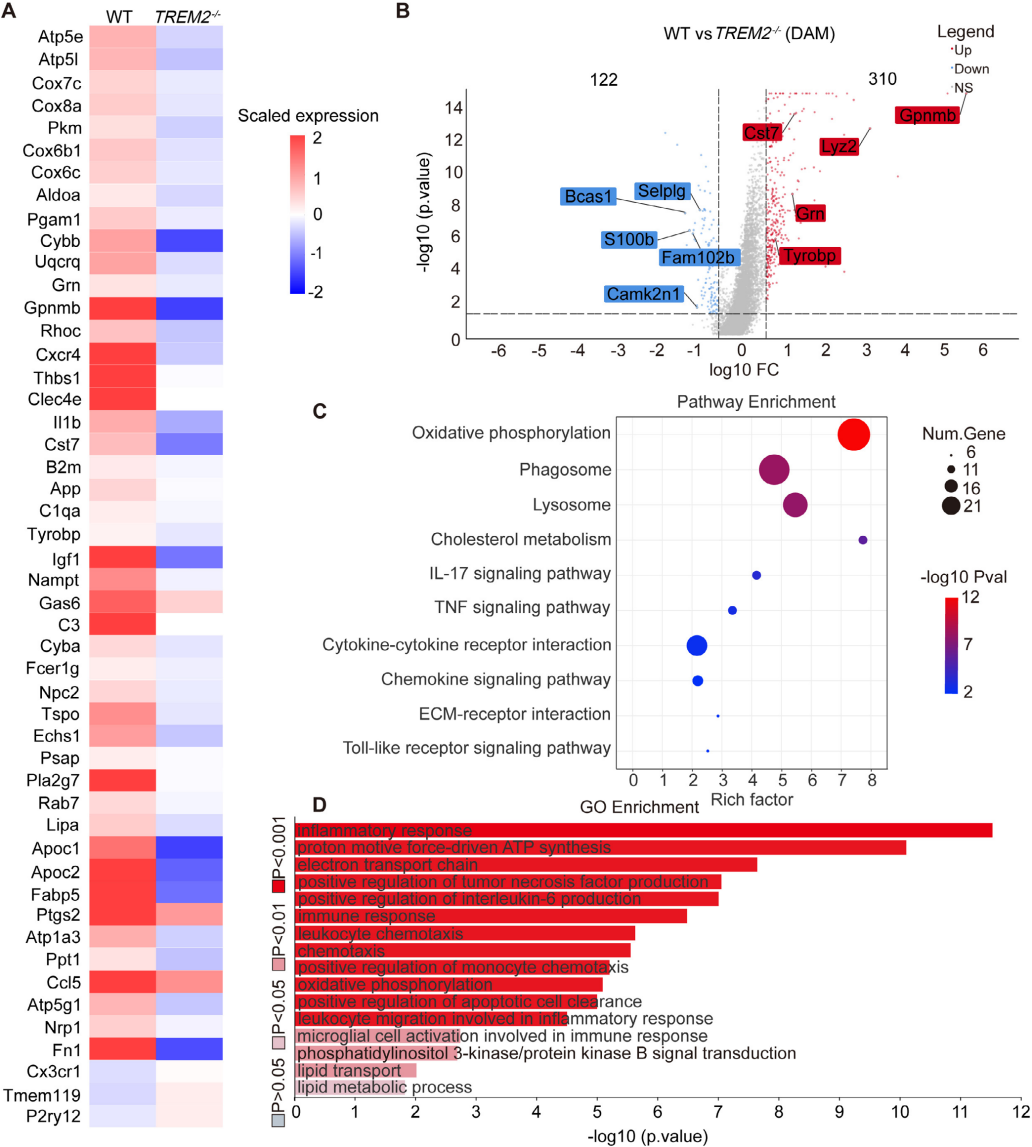
ScRNA‐Seq data analysis of WT and TREM2‐deficient DAM at 7 dpi. (A) Heatmap of differentially expressed genes (DEGs) involved in oxidative phosphorylation, phagosome, lysosome, cholesterol homeostasis, and phosphatidylinositol 3‐kinase/protein kinase B (PI3K/AKT) signal transduction (FDR < 0.1, FC > 1.5). (B) Volcano plot illustrating DEGs between WT and TREM2‐deficient DAM (FDR < 0.1, FC > 1.5). (C) Kyoto Encyclopedia of Genes and Genomes (KEGG) pathway enrichment bubble plot highlighting major pathways that are repressed in TREM2‐deficient DAM compared to WT DAM. The bubble size reflects the number of genes in each pathway, and the color scale indicates statistical significance (−log_10_ (*p*‐value)). (D) Gene ontology (GO) analysis bar graph highlighting significantly enriched biological processes, molecular functions, and cellular components that are repressed in TREM2‐deficient DAM compared to WT DAM. Color scale indicates statistical significance (−log_10_ (*p*‐value)).

To characterize the DAM cluster post‐injury, we analyzed the public single‐cell dataset GSE198852 [13] and observed 310 downregulated and 122 upregulated genes (FDR < 0.1) in TREM2‐deficient DAM compared to WT DAM at 7 dpi (Figure 5A,B). KEGG analysis further revealed that many of the downregulated genes in TREM2‐deficient DAM were associated with oxidative phosphorylation, phagosome, lysosome, cholesterol homeostasis, IL‐17 signaling pathways, tumor necrosis factor (TNF) signaling pathways, and cytokine‐cytokine receptor interactions (Figure 5C). Similarly, GO analysis showed significant repression of biological processes and molecular functions associated with energy metabolism, lipid metabolism, cell activation, inflammatory response, immune response, and positive regulation of TNF production in TREM2‐deficient DAM compared to WT DAM (Figure 5D). A previous study has proved that TREM2 promotes the PI3K/AKT pathway during the acute stage of TBI, enhances glycolysis in microglia, and partially facilitates the transformation into DAM‐like cells [33]. Similarly, the PI3K/AKT pathway was repressed in TREM2‐deficient DAM after SCI (Figure 5D), providing a mechanistic explanation for how TREM2 deficiency prevents the acquisition of a complete DAM phenotype following SCI. These findings suggest that TREM2 deficiency may hinder excessive DAM activation in response to myelin debris.

**FIGURE 6:**
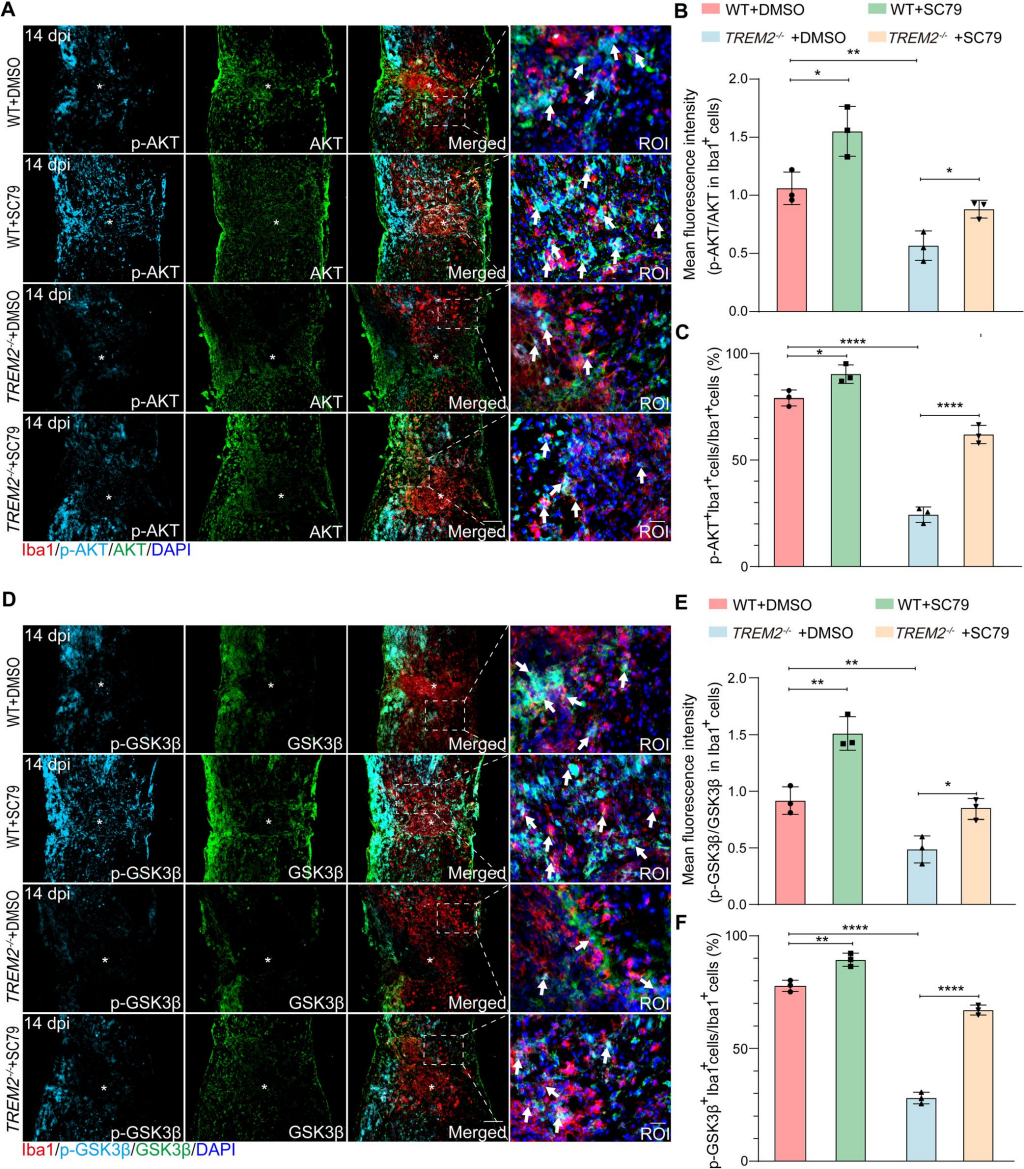
The administration of SC79 activates the PI3K/AKT pathway in microglia after SCI. (A) Representative images (co‐stained for p‐AKT, AKT, and Iba1) in the WT+DMSO, WT+SC79, *TREM2* ^−/−^+DMSO, and *TREM2* ^−/−^+SC79 groups at 14 dpi. Scale bars: Low magnification, 100 μm; high magnification, 20 μm. (B) As shown in (A), the ratio of mean fluorescence intensity (MFI) of p‐AKT to AKT in Iba1^+^ cells around the lesion core was quantified. (C) Percentage of Iba1^+^p‐AKT^+^ cells among total Iba1^+^ cells, as shown in (A). (D) Representative images (co‐stained for p‐GSK3β, GSK3β, and Iba1) in the WT+DMSO, WT+SC79, *TREM2* ^−/−^+DMSO, and *TREM2* ^−/−^+SC79 groups at 14 dpi. Scale bars: Low magnification, 100 μm; high magnification, 20 μm. (E) As shown in (D), the ratio of MFI of p‐GSK3β to GSK3β in Iba1^+^ cells around the lesion core was quantified. (F) Percentage of Iba1^+^p‐GSK3β^+^ cells among total Iba1^+^ cells, as shown in (D). Statistical significance between experimental groups was calculated by one‐way ANOVA followed by Tukey's post hoc test (B, C, E, F). *p < 0.05, **p < 0.01, ****p < 0.0001. Data are presented as mean ± SEM; each point represents an individual mouse.

Therefore, we conducted in vivo rescue experiments to investigate the role of the PI3K/AKT pathway in microglial activation following SCI. SC79 (dissolved in 5% DMSO/saline) was administered intraperitoneally (WT and *TREM2* ^−/−^ mice) at 0.5 h before SCI and then once daily after injury to induce AKT phosphorylation in microglia, while control groups (WT and *TREM2* ^−/−^ mice) received 5% DMSO/saline at equivalent volumes. At 14 dpi, quantification of Iba1^+^ microglia outside the lesion core revealed markedly reduced p‐AKT/AKT and p‐GSK3β/GSK3β ratios in the *TREM2* ^−/−^+DMSO group compared with the WT+DMSO group, indicating PI3K/AKT pathway suppression in the absence of TREM2 (Figure 6A,B,D,E). Notably, administration of SC79 significantly increased both p‐AKT/AKT and p‐GSK3β/GSK3β ratios in the *TREM2* ^−/−^+SC79 group relative to the *TREM2* ^−/−^+DMSO group, demonstrating that SC79 effectively restored the PI3K/AKT pathway in microglia under TREM2‐deficient conditions after SCI (Figure 6A,B,D,E). Consistent with these changes, the proportion of p‐AKT^+^Iba1^+^ and p‐GSK3β^+^Iba1^+^ microglia exhibited a similar trend across groups, further confirming the microglial PI3K/AKT pathway reactivation by SC79 after SCI (Figure 6A,C,D,F). Immunostaining at 14 dpi revealed a higher number of Stage 2 DAM in the WT+SC79 group compared to the WT+DMSO group (Figure S3A–D), suggesting that AKT phosphorylation promotes DAM activation. Similarly, more Stage 2 DAM were observed in the *TREM2* ^−/−^+SC79 group than in the *TREM2* ^−/−^+DMSO group (Figure S3A–D), indicating that AKT phosphorylation can partially rescue the impaired DAM activation resulting from TREM2 deficiency. Collectively, these findings strongly support the hypothesis that TREM2 promotes DAM activation via enhancing the PI3K/AKT pathway following SCI.

### 3.5 Inhibition of Inflammation and Fibrosis After SCI Requires TREM2 Deletion

**FIGURE 7:**
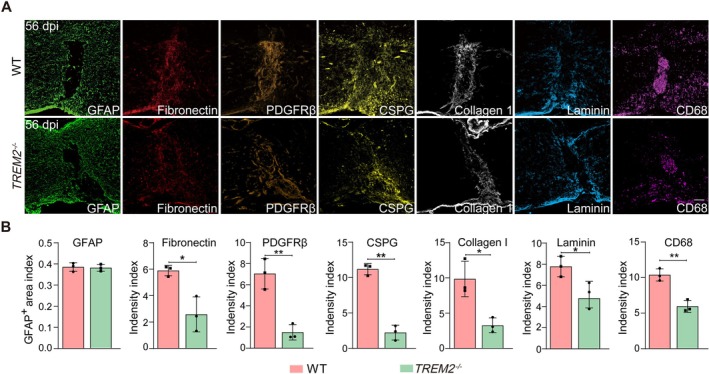
TREM2 deficiency alleviates inflammation and fibrosis after SCI. (A) Immunostaining of GFAP, Fibronectin, PDGFRβ, CSPG, collagen I, laminin, and CD68 at the lesion core at 56 dpi in WT and *TREM2* ^−/−^ mice. Scale bars: 100 μm. (B) Quantification of immunoreactive intensity for (A), normalized to the intact region. Statistical significance between experimental groups was calculated by unpaired Student's *t*‐test (B). *p < 0.05, **p < 0.01. Data are presented as mean ± SEM; each point represents an individual mouse.

The activation of DAM after SCI may contribute to persistent inflammation, which can promote fibrosis in the chronically injured spinal cord [34]. We next compared the pathological characteristics of lesion sites in WT and *TREM2* ^−/−^ mice at 56 dpi. In WT mice, the injury site exhibited a prominent accumulation of GFAP^+^ astrocytes surrounding the lesion core, a marked accumulation of PDGFRβ^+^ fibroblasts, deposition of ECM components (fibronectin, CSPG, collagen I, and laminin), and accumulation of CD68^+^ cells at the lesion core (Figure 7A,B). These results align with previous studies [35, 36]. By contrast, in *TREM2* ^−/−^ mice, a less organized scar structure had formed at the lesion core, with a significant decrease in the accumulation of fibroblasts, the deposition of ECM, and the accumulation of CD68^+^ cells (Figure 7A,B). However, the accumulation of GFAP^+^ astrocytes was similar between WT and *TREM2* ^−/−^ mice (Figure 7A,B). Together, these results demonstrate that TREM2 exacerbates inflammation and fibrosis in chronic SCI.

### 3.6 COG1410‐Mediated TREM2 Activation Is Involved in Regulating Locomotor Functional Recovery, Axon Regeneration, and Neuronal Survival After SCI

**FIGURE 8:**
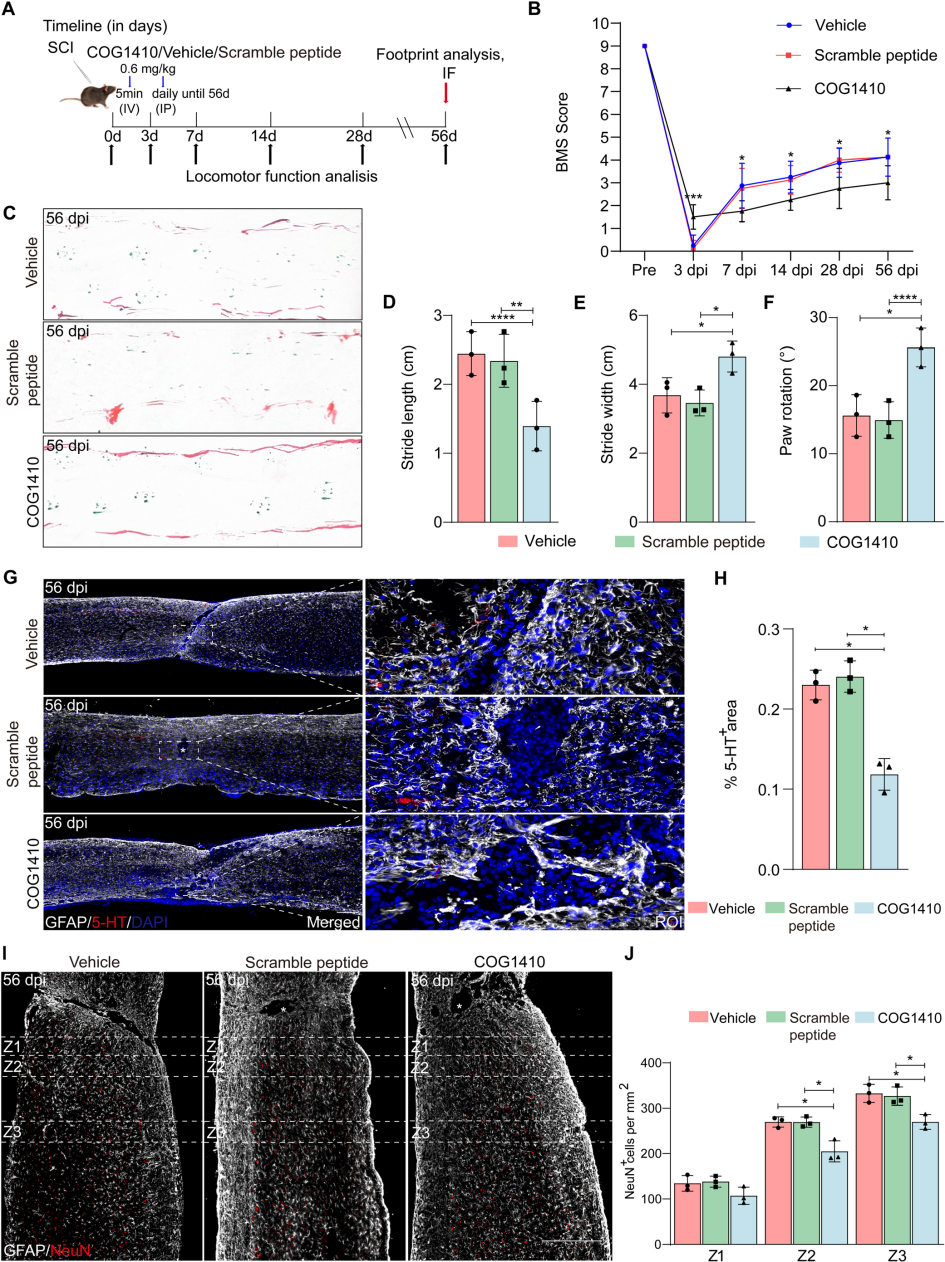
Continuous COG1410 treatment hinders axon regeneration and neuronal survival after SCI. (A) Schedule of COG1410 administration (i.v. and i.p.) and behavioral assessment. (B) Locomotor function was evaluated by BMS pre‐operation and at 3, 7, 14, 28, and 56 dpi. (C) Representative footprint analysis images from the vehicle group, the scramble peptide group, and the COG1410‐treated group at 56 dpi. The front paws are shown in green dyes, and the hind paws are shown in red dyes. (D–F) Quantification of the stride length, stride width, and paw rotation at 56 dpi. (G) Immunostaining of 5‐HT (red) and GFAP (white) in sagittal sections of all groups at 56 dpi. Scale bars: Low magnification, 200 μm; high magnification, 20 μm. (H) Quantification of the 5‐HT^+^ area of the spinal cord segment at 56 dpi. (I) Immunostaining of NeuN (red) and GFAP (white) in sagittal sections of all groups at 56 dpi. Scale bars: 200 μm. (J) Quantification of the number of NeuN in the caudal side of the spinal cord segment at 56 dpi. Statistical significance between experimental groups was calculated by two‐way ANOVA followed by Tukey's post hoc test (B) and (J), one‐way ANOVA followed by Tukey's post hoc test (D–F, H). *p < 0.05, **p < 0.01, ***p < 0.001, ****p < 0.0001. Data are presented as mean ± SEM; each point represents an individual mouse.

To investigate the role of TREM2 activation in locomotor recovery and axonal regeneration after SCI, mice received a single dose (I.V.) of COG1410 at 5 min post‐injury, followed by daily injections (I.P.) for up to 56 dpi (Figure 8A). After intervention with the TREM2 activator COG1410, we detected its activation efficiency on the TREM2 pathway and analyzed its impact on neuroinflammation. At 14 dpi, COG1410‐treated mice showed markedly enhanced activation of the PI3K/AKT signaling pathway compared with the vehicle and scrambled‐peptide groups (Figure S4A–C). The expression of the neuroinflammatory marker CST7 was also increased following COG1410 administration (Figure S4A,C). Locomotor function was evaluated through the BMS scores and footprint analysis [21, 22]. COG1410 treatment significantly improved BMS scores at 3 dpi compared to the vehicle and scramble peptide groups; however, scores subsequently declined and remained below those of the two control groups from 7 to 56 dpi (Figure 8B). Furthermore, the footprint analysis at 56 dpi revealed similar findings. Specifically, the COG1410‐treated group exhibited worse performance in hind limb function, including shorter step lengths, longer step widths, and bigger rotation angles (Figure 8C–F). These results suggest that sustained TREM2 activation hinders locomotor function recovery after SCI.

Locomotor function recovery is closely linked to enhanced axonal regeneration after SCI [37]. To explore the impact of TREM2 activation on axonal regeneration after SCI, we assessed the regeneration of 5‐HT^+^ axons using immunostaining at 56 dpi. COG1410 treatment significantly reduced the area of 5‐HT^+^ axons at the lesion site. Furthermore, fewer 5‐HT^+^ axon fibers were observed to penetrate the lesion core compared to the vehicle and scramble peptide groups (Figure 8G,H). Additionally, we assessed residual neurons in regions adjacent to the lesion core (Z1–Z3) using the neuronal marker NeuN [38]. The COG1410‐treated group showed a significant decrease in the number of residual NeuN^+^ neurons in the Z2–Z3 regions at 56 dpi, compared to the two control groups (Figure 8I,J). Collectively, these findings indicate that sustained TREM2 activation impairs both axonal regeneration and neuronal survival following SCI.

To investigate the role of short‐term TREM2 activation in locomotor function recovery and axonal regeneration after SCI, mice received a single dose (I.V.) of COG1410 at 5 min post‐injury, followed by daily injections (I.P.) until 3 dpi (Figure S5A). The results show that short‐term COG1410 treatment improved BMS scores at 3 dpi and produced a modest functional advantage at 7 dpi compared to the vehicle and scramble peptide groups (Figure S5B). However, this effect was transient and not maintained at later time points, with no significant differences observed among the groups by 56 dpi (Figure S5B). Consistent with these results, footprint analysis at 56 dpi showed no significant differences in stride length, stride width, or paw rotation between the short‐term COG1410 group and either control group (Figure S5C–F). These results indicate that short‐term TREM2 activation confers only early improvement in locomotor functional recovery. Additionally, short‐term COG1410 treatment failed to increase the area of 5‐HT^+^ axons at the lesion site (Figure S5G,H). Likewise, the number of residual NeuN^+^ neurons in the Z1‐Z3 regions at 56 dpi did not differ significantly between the short‐term group and either control group (Figure S5I,J). Collectively, these findings indicate that short‐term TREM2 activation promotes transient functional improvement without driving axonal regeneration and neuronal survival following SCI.

## 4 Discussion

This study demonstrates that TREM2 is a critical regulator of microglial responses to myelin debris. We found that TREM2 expression increased after SCI, with predominant expression in macrophages/microglia, particularly in microglia. TREM2 promoted the engulfment and degradation of myelin debris, thereby reducing its accumulation and foamy macrophage formation. However, TREM2 promoted DAM activation by activating the PI3K/AKT pathway and exacerbated fibrosis in the injured spinal cord. Furthermore, long‐term systemic activation of TREM2 by COG1410 impaired locomotor functional recovery, axonal regeneration, and neuronal survival following SCI, while short‐term activation facilitated early functional benefit without contributing to axonal regeneration or neuronal survival. Therefore, TREM2 is a key regulator that mediates myelin debris clearance, chronic inflammation, and fibrosis following SCI.

The accumulation of myelin debris following SCI represents a significant barrier to tissue repair [1, 2]. Following SCI, myelin debris accumulates during the initial week and remains present in the chronic SCI tissue for up to 1 year, inhibiting axon regeneration while triggering chronic inflammation, which further exacerbates tissue damage [39]. In this study, we observed that TREM2 expression was upregulated during the active phagocytic phase (3–7 dpi) [40] of macrophages/microglia after SCI. This temporal pattern implies that TREM2 may be essential in the engulfment of myelin debris. Indeed, TREM2 deficiency resulted in a significant reduction in myelin debris engulfment by microglia, as evidenced by decreased levels of dMBP/MBP within microglia. This observation aligns with a prior study indicating that TREM2 deficiency reduces the phagocytic phenotype of microglia after SCI [13]. Additionally, Rong Limin's group utilized the T8 contusion model in their study [13]. Considering the complexity of human SCI, we selected the T10 lateral crush model, which differs from their approach. Impaired myelin debris clearance can lead to foamy macrophage formation, characterized by the accumulation of lipid droplets after SCI [3, 39]. In conditions of TREM2 deficiency, we detected a notable elevation in Oil Red O staining or BODIPY staining, indicating enhanced lipid accumulation within macrophages/microglia. This suggests that TREM2 is not only involved in the initial engulfment of myelin debris but also in the subsequent degradation of lipids. By coordinating phagocytic clearance with intracellular cholesterol processing and efflux, TREM2 ensures that myelin‐derived lipids are efficiently recycled rather than retained, thereby facilitating axonal regeneration to some extent [41, 42]. The inability of TREM2‐deficient macrophages/microglia to effectively metabolize myelin‐derived lipids contributes to the persistence of foamy macrophages, which may impair behavioral and histological outcomes after injury [1, 3, 24]. Collectively, our findings suggest that TREM2 is crucial for both myelin debris clearance and the prevention of foam cell formation.

Microglia are the primary immune cells in the CNS and are rapidly activated following injury [43, 44, 45]. Under normal conditions, microglia maintain a homeostatic state, characterized by the expression of markers such as P2ry12. However, following SCI, microglia undergo a phenotypic shift to become DAM, which is characterized by the upregulation of markers such as CST7 and IGF1 [7]. As a distinct microglial population, DAM participates in various processes such as the engulfment and degradation of myelin debris [7, 26, 46, 47, 48, 49]. However, DAM is also predicted to be involved in cytokine production, ROS response, and cell death regulation, suggesting that DAM may continue to sustain a pro‐inflammatory microenvironment in the chronic SCI [50]. The reduced expression of DAM markers in TREM2‐deficient microglia indicates that TREM2 is essential for the transcriptional reprogramming that drives the transformation into the DAM phenotype. Dataset GSE198852 revealed that, compared to TREM2‐deficient DAM, WT DAM not only upregulate key genes involved in debris clearance (e.g., positive regulation of phagocytosis and lysosome activity), lipid metabolism (particularly cholesterol metabolism), but also aberrantly activate pathways associated with pro‐inflammatory effects, including cell activation involved in inflammation response, IL‐17 signaling, TNF signaling, and cytokine‐cytokine receptor interactions. Moreover, dataset GSE198852 further revealed that TREM2 deficiency impairs the PI3K/AKT pathway in DAM, a pathway that is known to be critical for microglial activation [13]. The PI3K/AKT pathway participates in regulating phagocytosis and glucose metabolism, including processes such as oxidative phosphorylation and glycolysis [33], and its repression in TREM2‐deficient microglia likely contributes to the impaired transformation into the DAM phenotype. Therefore, it is worth further exploring the specific regulatory mechanism of TREM2 in the PI3K/AKT pathway and examining the impact of the TREM2/PI3K/AKT signaling axis on the SCI microenvironment. We further demonstrated that systemic administration of the AKT activator SC79 partially restored the impaired DAM activation in *TREM2* ^−/−^ mice after SCI. Taken together, these findings support the hypothesis that TREM2 promotes DAM activation through activating the PI3K/AKT pathway. In this study, we found that TREM2 facilitated the clearance of myelin debris by microglia after SCI, but it also promoted DAM activation via activating the PI3K/AKT pathway, leading to chronic inflammation and fibrosis. These findings further support the notion that DAM may exert pro‐inflammatory effects following SCI. To more precisely verify the role of DAM in SCI pathology, we need to employ genetic manipulation or pharmacological approaches to specifically deplete this microglial subset in future studies.

Persistent inflammation exacerbates fibrosis and neuronal cell death at the lesion site [3]. Fibrosis is a hallmark of chronic SCI, characterized by excessive deposition of ECM components, which forms a barrier that impedes axonal regeneration and locomotor function recovery. The alleviated fibrosis observed in *TREM2* ^−/−^ mice is likely linked to limited DAM activation rather than being the direct consequence of TREM2 clearance, which is inferred based on our finding of low TREM2 expression in PDGFRβ^+^ fibroblasts. In future studies, microglia‐specific and macrophage‐specific TREM2 knockout mice should be used to explore the effects of TREM2‐mediated microglia/macrophage phagocytosis on the scar microenvironment. Additionally, the fibrosis‐suppressing effect observed in TREM2‐deficient mice after SCI likely involves coordinated multicellular responses, and further mechanistic studies will be required. Based on existing evidence, TREM2 deficiency may attenuate astrocyte activation, thereby further reducing pathological ECM deposition and constraining fibrosis progression after SCI [51].

Repairing SCI continues to be a major challenge, with two promising strategies emerging as potential solutions. The first involves epidural electrical stimulation (EES) using biocompatible electrodes and brain‐computer interfaces to bypass glial scars [52], as demonstrated by Grégoire Courtine's group at EPFL, who developed personalized lumbar implants to activate Vsx2^+^ and Hoxa10^+^ neurons and a brain‐spinal interface for cortical signal modulation [53, 54]. However, optimizing EES parameters, enhancing biocompatibility, and expanding clinical trials are needed. The second strategy employs biological approaches, such as growth factor delivery and biomaterials, to inhibit scar formation and promote axon regeneration [55, 56]. Sofroniew MV's group at UCLA utilized AAV‐mediated overexpression of certain growth factors in thoracic neurons of the spinal cord and collaborated with other groups on a hydrogel containing a variety of nutrient factors to establish a “push‐pull” system to guide axon regeneration to the lumbar spinal cord [56, 57]. This technology also requires further clinical translation. Our previous studies demonstrated that blocking the PDGFRβ pathway with SU16f and imatinib directly inhibited fibrous scar formation [25, 58]. In future research, we aim to integrate this direct inhibition with other indirect effects, developing a combination therapy akin to that proposed by Professor Sofroniew MV to enhance axon regeneration [56, 57].

In this study, young female mice were used to minimize the risk of urinary tract infections associated with voiding dysfunction. However, gender and age variables were not analyzed, which may restrict the broader applicability of our findings. While previous SCI studies have predominantly utilized female models [41, 59, 60, 61, 62], the known gender differences in microglial function and the gender‐balanced distribution of clinical patients highlight the importance of considering gender as a factor in both pathological and therapeutic mechanisms [60, 63]. Future studies incorporating male and aged animal models, along with age‐stratified human data, will be essential to validate the applicability of TREM2 regulatory mechanisms across diverse populations. In addition, all transgenic mouse lines used in this study were validated by PCR‐based genotyping in accordance with the provider's protocol, with representative results shown in Figure S6A–D.

## 5 Conclusions

In conclusion, our study demonstrates that TREM2 plays a critical role in SCI pathology: it facilitates myelin debris clearance but also exacerbates chronic inflammation and fibrosis. Notably, sustained TREM2 activation impairs locomotor functional recovery, axonal regeneration, and neuronal survival, whereas transient activation offers limited early functional benefit without supporting axonal regeneration or neuronal survival. These findings provide a foundation for the development of TREM2‐targeted pharmacological or gene therapy strategies to enhance recovery after SCI.

## Author Contributions

Li Cheng, Juehua Jing, and Dasheng Tian designed the study. Zhonghan Wu, Shuisheng Yu, and Yixue Hu performed the experiments and analyzed the data. Zhonghan Wu was a major contributor in writing the manuscript with participation from other authors. Li Cheng, Juehua Jing, Dasheng Tian, and Shuisheng Yu provided funding. All authors read and approved the final manuscript.

## Funding

This study was supported by the National Natural Science Foundation of China (Grant number 82271413, 82401616), Anhui Provincial Clinical Research Transformation Project (Grant number 202304295107020013, 202304295107020009, 202304295107020011), Natural Science Foundation of Anhui Province (2408085QH262), and Natural Science Research Key Project of Colleges and Universities of Anhui Province (Grant number: KJ2021A0310).

## Ethics Statement

Animal experiments were approved by the Animal Ethics Committee of Anhui Medical University (approval number: LLSC20232105).

## Conflicts of Interest

The authors declare no conflicts of interest.

## Supporting information


**Data S1:** Supplementry methods.
**Figure S1:** TREM2 knockdown efficiency in primary microglia and the effect of myelin debris treatment on TREM2 expression.
**Figure S2:** TREM2 deficiency prevents the continued presence of DAM.
**Figure S3:** The administration of SC79 partially restores the impaired DAM activation in *TREM2* ^−/−^ mice after SCI.
**Figure S4:** COG1410 activates the TREM2‐mediated PI3K/AKT pathway and upregulates the neuroinflammatory marker CST7.
**Figure S5:** Short‐term COG1410 treatment improves early locomotor function recovery but fails to drive axon regeneration or neuronal survival after SCI.
**Figure S6:** Genotyping of transgenic mice lines by PCR and sequencing.

## Data Availability

The data that support the findings of this study are available from the corresponding author upon reasonable request.
