# Supplementary material for: TREM2 Facilitates Myelin Debris Clearance but Exacerbates Chronic Inflammation and Fibrosis After Spinal Cord Injury
Source: CNS Neurosci Ther. 2026 Feb 9;32(2):e70777. doi: 10.1002/cns.70777 (PMC12884443; doi:10.1002/cns.70777)
Supplement: Supplementary file 1 — Data S1: Supplementry methods. Figure S1: TREM2 knockdown efficiency in primary microglia and the effect of myelin debris treatment on TREM2 expression. Figure S2: TREM2 deficiency prevents the continued presence of DAM. Figure S3: The administration of SC79 partially restores the impaired DAM activation in TREM2 −/− mice after SCI. Figure S4: COG1410 activates the TREM2‐mediated PI3K/AKT pathway and upregulates the neuroinflammatory marker CST7. Figure S5: Short‐term COG1410 treatment improves early locomotor function recovery but fails to drive axon regeneration or neuronal survival after SCI. Figure S6: Genotyping of transgenic mice lines by PCR and sequencing. [file CNS-32-e70777-s001.docx]

Supplementary Materials for

**TREM2 facilitates myelin debris clearance but exacerbates chronic inflammation and fibrosis after spinal cord injury**

Zhonghan Wu et al.

Corresponding author: Li Cheng, chengli7788@163.com

**Materials include:**

Supplementary Figures 1-6

Legends for Supplementary Figures 1-6

Supplementary Methods


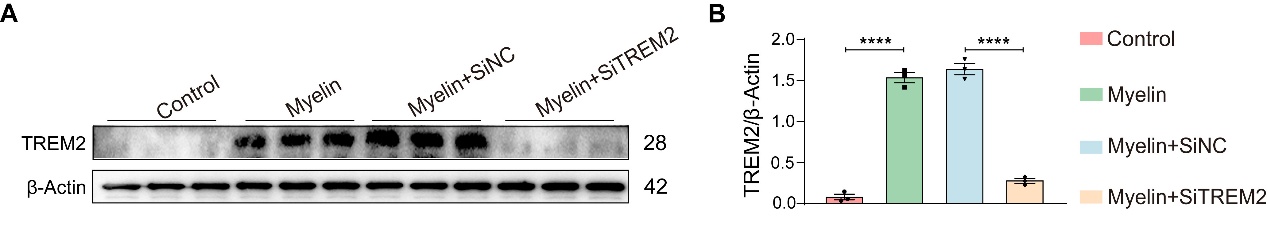


**Supplementary Figure 1 TREM2 knockdown efficiency in primary microglia and the effect of myelin debris treatment on TREM2 expression**

Primary microglia were first transfected with a negative control siRNA (SiNC) or a TREM2-targeting siRNA (SiTREM2); 24 hours later, subsets of transfected/untransfected microglia were treated with myelin debris (as indicated in each group). Western blotting was performed 24 hours post-myelin treatment.

**A** Representative western blotting images showing TREM2 expression under different treatments: untreated control, Myelin (untransfected+myelin), Myelin+SiNC (SiNC-transfected+myelin), and Myelin+SiTREM2 (SiTREM2-transfected+myelin); β-Actin was used as the loading control.

**B** Quantification of TREM2/β-Actin ratios corresponding to (**A**).

Statistical significance between experimental groups was calculated by one-way ANOVA followed by Tukey’s post hoc test (**B**). ^****^*P* < 0.0001. Data are presented as mean ± SEM; each point represents an independent culture.


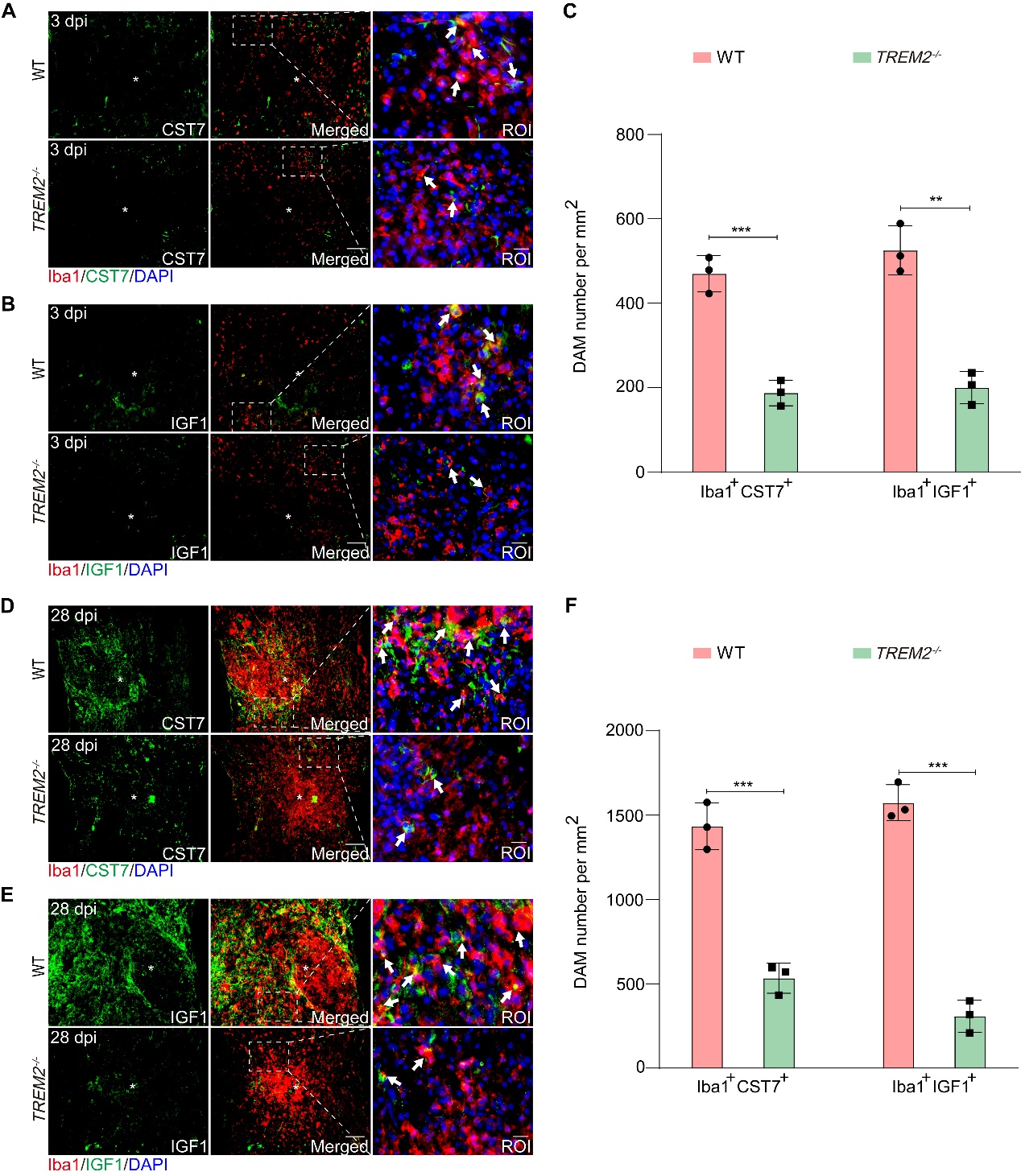


**Supplementary Figure 2 TREM2 deficiency prevents the continued presence of DAM.**

**A, B** Microglia show lower expression of CST7 and IGF1 in *TREM2^-/-^* mice than in WT mice at 3 dpi. Scale bars: low magnification, 100 μm; high magnification, 20 μm.

**C** Quantification of Iba1^+^CST7^+^ and Iba1^+^IGF1^+^ DAM number in WT and *TREM2^-/-^* mice at 3 dpi.

**D, E** Microglia show lower expression of CST7 and IGF1 in *TREM2^-/-^* mice than in WT mice at 28 dpi. Scale bars: low magnification, 100 μm; high magnification, 20 μm.

**F** Quantification of Iba1^+^CST7^+^ and Iba1^+^IGF1^+^ DAM number in WT and *TREM2^-/-^* mice at 28 dpi.

Statistical significance between experimental groups was calculated by unpaired Student’s t-test (**C**) and (**F**). ^**^ *P* < 0.01, ^***^ *P* < 0.001. Data are presented as mean ± SEM; each point represents an individual mouse.


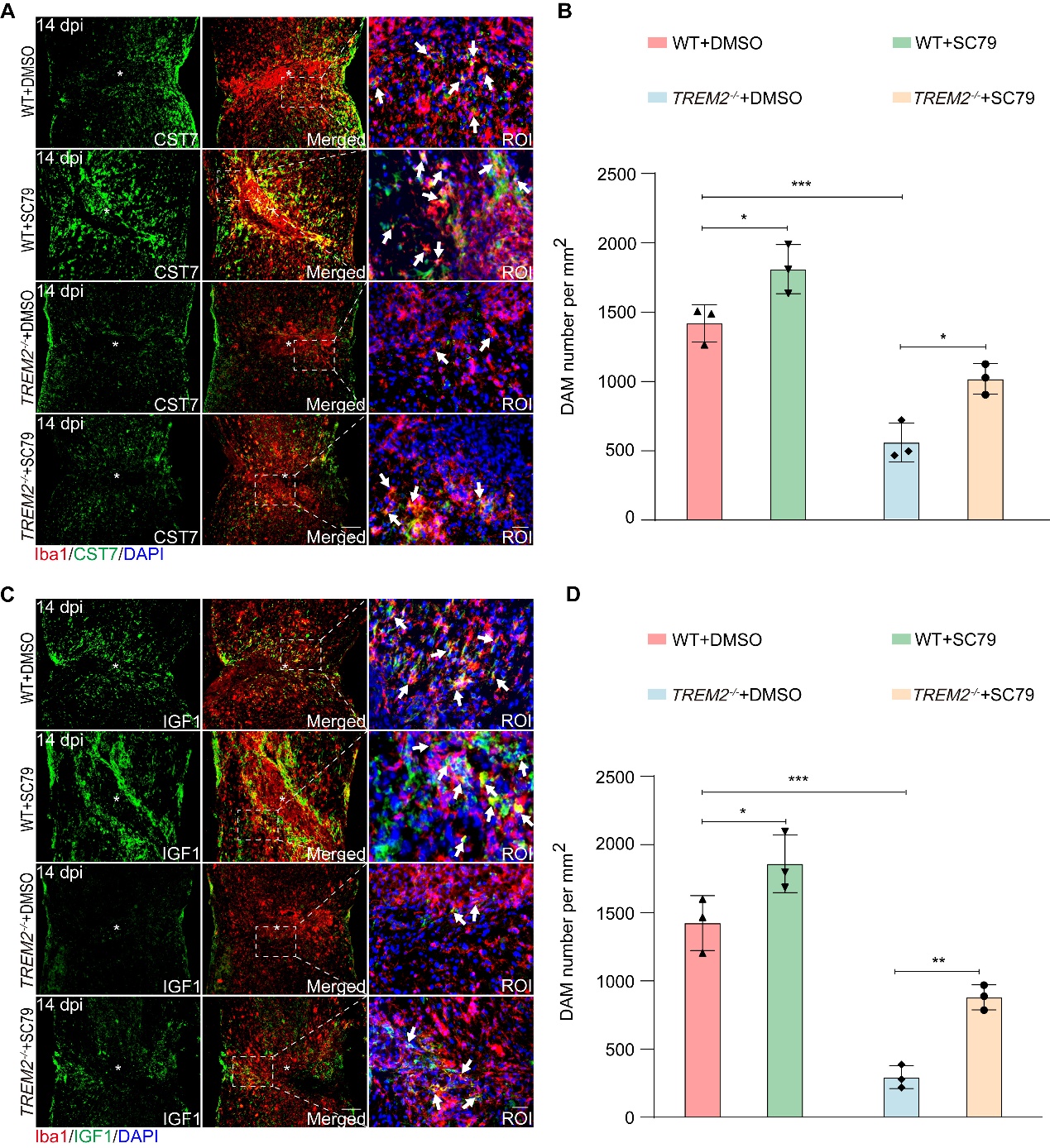


**Supplementary Figure 3 The administration of SC79 partially restores the impaired DAM activation in *TREM2^-/-^* mice after SCI.**

**A** Microglia show higher expression of CST7 in the WT+SC79 group than in the WT+DMSO group at 14 dpi. Microglia show higher expression of CST7 in *TREM2^-/-^*+SC79 group than in *TREM2^-/-^*+DMSO group at 14 dpi. Scale bars: low magnification, 100 μm; high magnification, 20 μm.

**B** Quantification of the Iba1^+^CST7^+^ DAM number in (**A**).

**C** Microglia show higher expression of IGF1 in the WT+SC79 group than in the WT+DMSO group at 14 dpi. Microglia show higher expression of IGF1 in *TREM2^-/-^*+SC79 group than in *TREM2^-/-^*+DMSO group at 14 dpi. Scale bars: low magnification, 100 μm; high magnification, 20 μm.

**D** Quantification of the Iba1^+^IGF1^+^ DAM number in (**C**).

Statistical significance between experimental groups was calculated by one-way ANOVA followed by Tukey’s post hoc test (**B**) and (**D**). ^*^*P* < 0.05, ^**^*P* < 0.01, ^***^*P* < 0.001. Data are presented as mean ± SEM; each point represents an individual mouse.


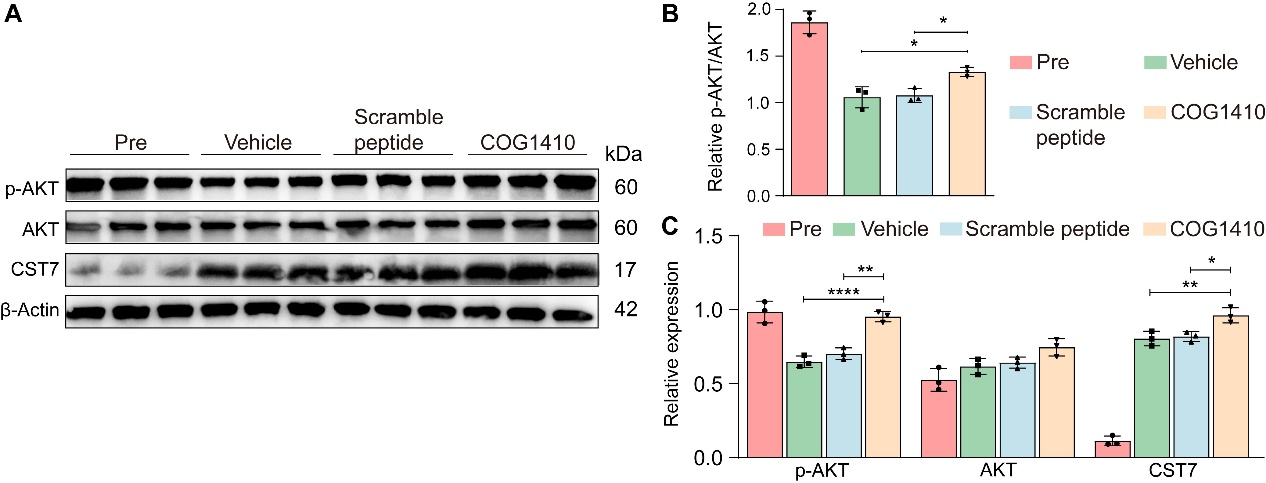


**Supplementary Figure 4 COG1410 activates the TREM2-mediated PI3K/AKT pathway and upregulates the neuroinflammatory marker CST7.**

**A** Representative western blotting bands depicting the expression of p-AKT, AKT, CST7, and β-Actin (as a loading control) in four groups: Pre (naive condition), Vehicle, Scramble peptide, and COG1410-treated at 14 dpi.

**B** Quantitative analysis of the densitometric ratio of p-AKT to total AKT.

**C** Relative expression levels of p-AKT, AKT, and CST7, normalized to β-Actin and compared across groups.

Statistical significance between experimental groups was calculated by one-way ANOVA followed by Tukey’s post hoc test (**B**) and (**C**). ^*^*P* < 0.05, ^**^*P* < 0.01, ^****^*P* < 0.0001. Data are presented as mean ± SEM; each point represents an individual mouse.


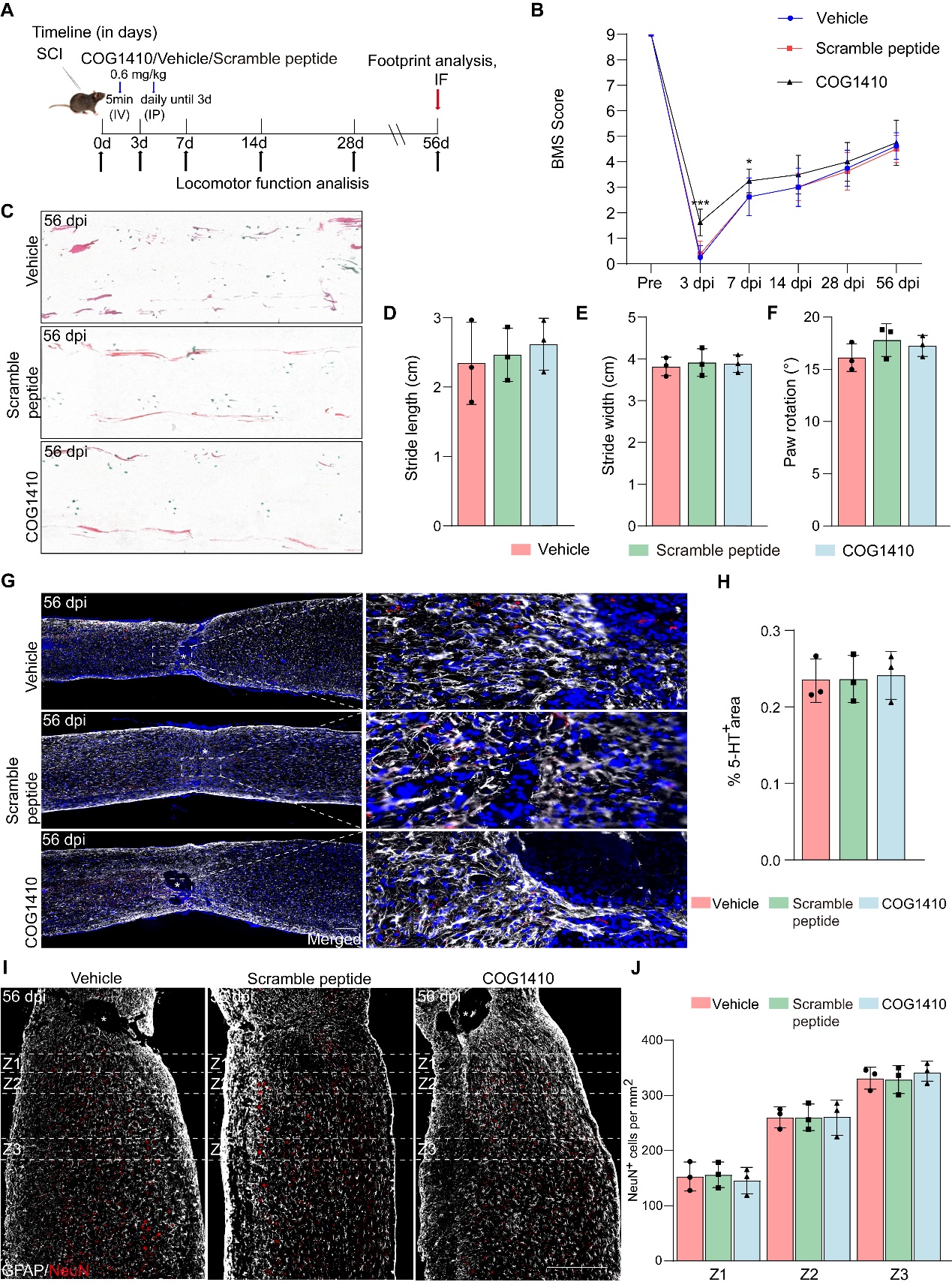


**Supplementary Figure 5 Short-term COG1410 treatment improves early locomotor function recovery but fails to drive axon regeneration or neuronal survival after SCI.**

**A** Schedule of COG1410 administration (i.v. and i.p.) and behavioral assessment.

**B** Locomotor function was evaluated by BMS pre-operation and at 3, 7, 14, 28, and 56 dpi.

**C** Representative footprint analysis images from the vehicle group, the scramble peptide group, and the COG1410-treated group at 56 dpi. The front paws are shown in green dyes, and the hind paws are shown in red dyes.

**D-F** Quantification of the stride length, stride width, and paw rotation at 56 dpi.

**G** Immunostaining of 5-HT (red) and GFAP (white) in sagittal sections of these three groups at 56 dpi. Scale bars: low magnification, 200 μm; high magnification, 20 μm.

**H** Quantification of the 5-HT^+^ area of the spinal cord segment at 56 dpi.

**I** Immunostaining of NeuN (red) and GFAP (white) in sagittal sections of these three groups at 56 dpi. Scale bars: 200 μm.

**J** Quantification of the number of NeuN in the caudal side of the spinal cord segment at 56 dpi.

Statistical significance between experimental groups was calculated by two-way ANOVA followed by Tukey’s post hoc test (**B**) and (**J**), one-way ANOVA followed by Tukey’s post hoc test (**D**), (**E**), (**F**), and (**H**). ^*^*P* < 0.05, ^***^*P* < 0.001. Data are presented as mean ± SEM; each point represents an individual mouse.


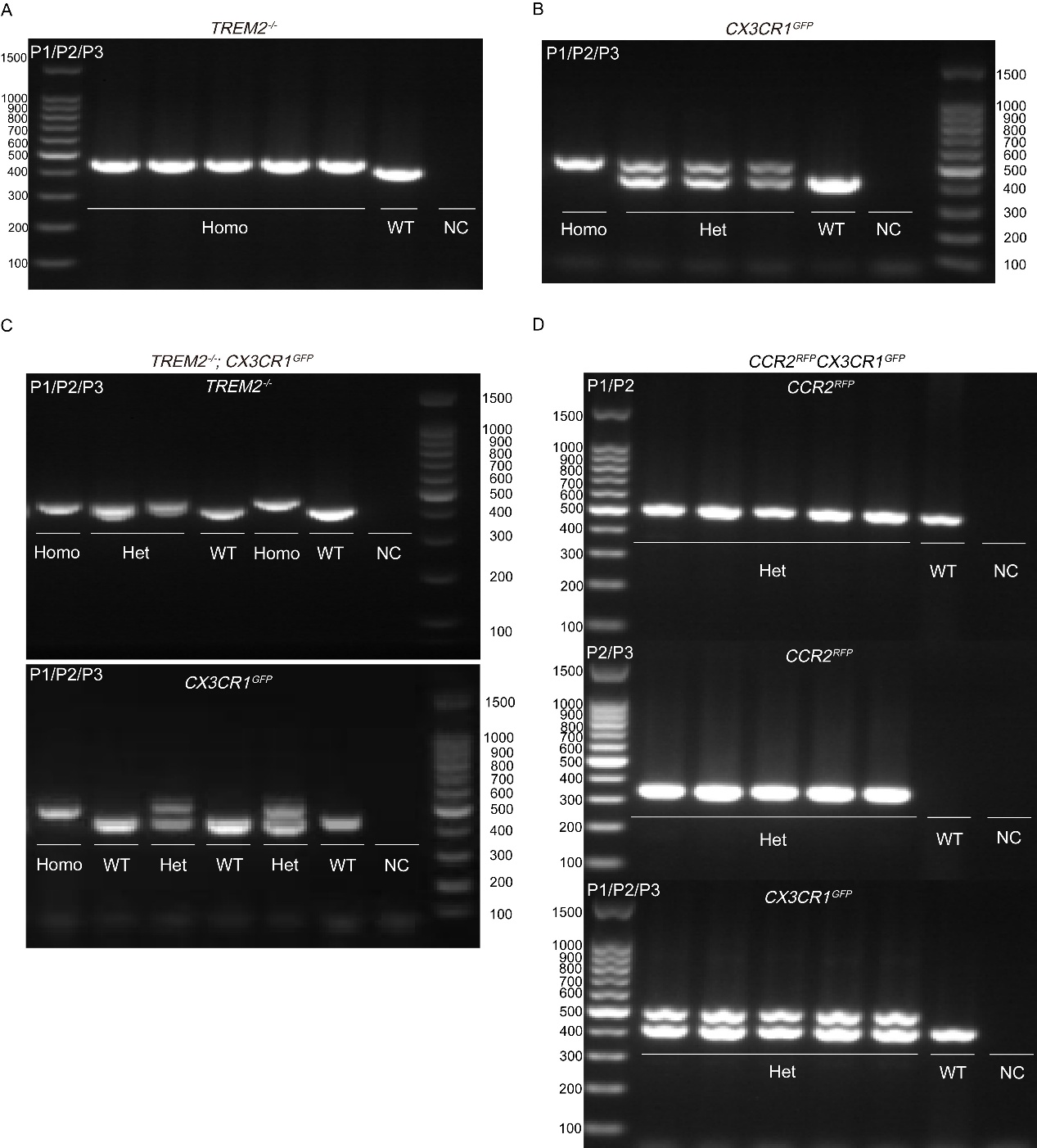


**Supplementary Figure 6 Genotyping of transgenic mice lines by PCR and Sequencing.**

**A** Genotyping of *TREM2^⁻/⁻^* mice using a three-primer PCR strategy (P1/P2/P3). Homozygous knockout (Homo), wild-type (WT), and negative control (NC) samples show the expected allele-specific bands.

**B** Genotyping of *CX3CR1^GFP^* mice using a three-primer PCR strategy (P1/P2/P3). Homozygous knock-in (Homo), heterozygous (Het), WT, and NC samples display the corresponding GFP knock-in and WT allele bands.

**C** Genotyping of *TREM2^⁻/⁻^*; *CX3CR1^GFP^* double-transgenic mice. Upper panel: TREM2 genotyping using P1/P2/P3 showing Homo, Het, and WT alleles; lower panel: *CX3CR1^GFP^* genotyping using P1/P2/P3 showing Homo, Het, and WT bands.

**D** Genotyping of *CCR2^RFP^CX3CR1^GFP^* dual-reporter mice. Top panel: WT-specific band for the CCR2 locus detected using P1/P2 (WT-specific); middle panel: Mutant (RFP)-specific band for the CCR2 locus detected using P2/P3 (mutant-specific); bottom panel: *CX3CR1^GFP^* genotyping using P1/P2/P3 showing Het and WT alleles. NC in all images indicates negative control.

**Supplementary methods**

***Phagocytosis-related images analysis***

Phagocytosis-related images were processed and analyzed using Imaris Software (Bitplane, Switzerland). For the quantification of degraded myelin basic protein (dMBP) engulfment by microglia, the dMBP fluorescence located in Iba1^+^ regions was included in the analysis. Similarly, for quantification of myelin basic protein (MBP) engulfment by microglia, the MBP fluorescence located in P2ry12^+^ regions was included in the analysis. A novel method for examining engulfed dMBP or engulfed MBP involved utilizing the mask function in Imaris to mask the dMBP fluorescence within Iba1^+^ regions or the MBP fluorescence within P2ry12^+^ regions. The volume of engulfed materials was quantified according to the previously published '3D Surface Rendering of Engulfed Material' protocol [1]. Considering differences in cell size, the volume of engulfed materials was normalized to the total volume of microglia per image field, as determined by the total volume of Iba1^+^ or P2ry12^+^ cells.

***Western blotting***

The total spinal cord protein was extracted with RIPA buffer (P0013B, Beyotime, China) supplemented with a Protease and phosphatase inhibitor cocktail (P1045, Beyotime, China). Protein concentration in all extracts was determined via bicinchoninic acid (BCA) assay to ensure uniform loading: Spinal cord protein samples were quantified using a BCA protein assay kit (P0010S, Beyotime, China). Equal amounts of protein were subjected to sodium dodecyl sulfate-polyacrylamide gel electrophoresis (SDS-PAGE) and transferred to polyvinylidene difluoride (PVDF) membranes. For spinal cord proteins: 20 μg of protein per sample was loaded onto SDS-PAGE gels for separation, followed by transfer to PVDF membranes. PVDF membranes were blocked by QuickBlock™ Blocking Buffer (P0220, Beyotime, China) at RT for 15 min to reduce non-specific antibody binding, with slight adjustments based on sample type. All primary antibodies were diluted in primary antibody dilution buffer (P0023A, Beyotime, China) and incubated with membranes at 4°C overnight. After washing with TBST, membranes were incubated with horseradish peroxidase (HRP)-conjugated secondary antibodies at RT for 1 h. Protein bands were visualized using enhanced chemiluminescence (ECL) detection reagents (P0018AS, Beyotime, China). All signals were imaged using the iBright™ FL1500 imaging system (A44241, Thermo Fisher Scientific, Waltham, MA, USA). Band gray values were quantified using ImageJ 2.0 software. Target protein expression levels were normalized to the corresponding loading control (β-actin) to correct for loading variability. The primary antibodies and secondary antibodies used are as follows: TREM2 (1:500, sc-373828, Santa Cruz, USA), CST7 (1:1000, PA5-103772, Thermo Fisher Scientific, Waltham, MA, USA), AKT (1:1000, MA5-41139, Thermo Fisher Scientific, Waltham, MA, USA), p-AKT (1:500, sc-514032, Santa Cruz, USA), β-Actin (1:5000, 60008-1-Ig, Proteintech, China), Goat anti-mouse IgG-HRP (1:10000; 31437, Thermo Fisher Scientific, Waltham, MA, USA), and Goat anti-rabbit IgG-HRP (1:10000; 31460, Thermo Fisher Scientific, Waltham, MA, USA).

***RNA-Seq Data Analysis:***

The public single-cell dataset analyzed in this study is available in the NCBI GEO repository under accession number [GSE198852] (https://www.ncbi.nlm.nih.gov/ geo/query/acc.cgi?acc=GSE198852). The data analysis for single-cell RNA sequencing (scRNA-seq) was carried out using the NovelBrain Cloud Analysis Platform (NovelBio Co., Ltd.). The raw sequencing reads were processed with fastp (default settings) to exclude adapter fragments and filter low-quality reads, resulting in preprocessed data. For the analysis of single-cell transcriptomes, UMI tools were utilized to identify the whitelist of cell barcodes. The high-quality UMI data were subsequently aligned with the mouse genome (Ensembl version 100) using STAR mapping, incorporating customized parameters from the UMI-tools standard pipeline to derive the UMI counts for each sample. This alignment facilitated the extraction of UMI counts for individual sample. Cells expressing more than 200 genes and exhibiting a mitochondrial UMI rate below 20% were retained for downstream analysis. Furthermore, Mitochondrial genes were subsequently excluded from the expression matrix. Differentially expressed genes (DEGs) were filtered using the DESeq2 algorithm [2]. Following the significance analysis, the P-value and False Discovery Rate (FDR) were evaluated according to these conditions: i) Fold Change (FC) > 1.5; ii) *P* < 0.05, FDR < 0.05 [3]. Heatmaps were generated by the analysis platform. Volcano plots were also generated by the analysis platform to visualize DEGs, applying a significance threshold of *P* < 0.05 and FC > 1.5. Pathway analysis was conducted to pinpoint the key pathways associated with the DEGs, utilizing the Kyoto Encyclopedia of Genes and Genomes (KEGG) database [4]. Key pathways were selected through Fisher’s exact test (*P* < 0.05). In addition, Gene Ontology (GO) analysis revealed the biological relevance of the DEGs identified in the experiment [5]. GO annotations were retrieved from UniProt, NCBI, and the Gene Ontology Consortium. Fisher’s exact test (*P* < 0.05) was used to identify the enriched GO terms. The KEGG analysis bubble plot and the GO analysis bar graph were also generated using this analysis platform.

***Genotyping of TREM2^-/-^, CX3CR1GFP, TREM2^-/-^; CX3CR1GFP and CCR2^RFP^CX3CR1^GFP^ Mice***

Genotyping for *TREM2^-/-^*, *CX3CR1^GFP^*, *TREM2^-/-^*; *CX3CR1^GFP^*, and *CCR2^RFP^CX3CR1^GFP^* mice was performed using genomic DNA isolated from tail tips. DNA was prepared using a standard lysis protocol and used directly for PCR amplification. Each genotype was determined by PCR using allele-specific primer sets, followed by agarose gel electrophoresis on 2% gels to distinguish wild-type, heterozygous, and homozygous alleles based on expected band sizes. A 100 bp DNA Ladder Marker (100-1500 bp; MB12666, MeilunBio, China) was used as the size standard to determine the molecular weight of PCR products. Representative electrophoresis images for all strains are provided in revised Supplementary Figure 6A-D.

Primer Sequences and Expected Band Sizes

***TREM2^⁻/⁻^***

P1 (WT-specific): TGCTCCCATTCCGCTTCTTC;

P2 (Common): CTCACGGGCAAGCTCATAGG;

P3 (Mutant-specific): TGGTTGGTGCACAGGTATGT

Expected bands: WT 415 bp; KO 449 bp; Het 415 + 449 bp

***CCR2^RFP^***

P1 (WT-specific): GGAGTAGAGTGGAGGCAGGA;

P2 (Common): TAAACCTGGTCACCACATGC;

P3 (Mutant-specific): CTTGATGACGTCCTCGGAG

Expected bands: WT 494 bp; RFP 320 bp; Het 494 + 320 bp

***CX3CR1^GFP^***

P1 (WT-specific): GTCTTCACGTTCGGTCTGGT;

P2 (Common): CCCAGACACTCGTTGTCCTT;

P3 (GFP-specific): CTCCCCCTGAACCTGAAAC

Expected bands: WT 350 bp; GFP 500 bp; Het 350 + 500 bp

***Primary Microglia Isolation, Culture, and siRNA Transfection***

Primary microglia were isolated from mixed-sex neonatal mice (postnatal days 1-4) using a standard protocol with slight modifications [6]. After removal of the olfactory bulb and cerebellum, brain tissue was minced in ice-cold PBS and transferred into 6-well plates. Samples were digested with 0.5 mL of enzymatic dissociation buffer per brain (C0203, Beyotime, China) at 37 °C for 20 min in a 5% CO₂ incubator. Cell suspensions were filtered through a 40-µm cell strainer and centrifuged at 200 × g for 10 min at room temperature. Pelleted cells were resuspended in DMEM (12100046, Gibco, USA), seeded into T25 or T75 flasks at approximately 5 × 10⁶ cells per flask, and cultured at 37 °C with 5% CO₂ until mixed glial cultures were established. LADMAC-conditioned medium was obtained by collecting the supernatant from LADMAC cells (CRL-2420, ATCC, USA), followed by centrifugation at 200 × g for 10 min and filtration through a 0.22-µm membrane. Primary microglia were maintained in DMEM supplemented with 20% LADMAC-conditioned medium, 10% fetal bovine serum (10270106, Gibco, USA), and 1% penicillin–streptomycin (C0222, Beyotime, China), under standard incubation conditions [6]. For gene silencing, microglia were transfected with either a TREM2-targeting siRNA (SiTREM2: sense 5′-GUACUUAUGACGCCUUGAATT-3′; antisense 5′-UUCAAGGCGUCAUAAGUACTT-3′) or a nonspecific control siRNA (SiNC: sense 5′-UUC UCC GAA CGU GUC ACG UTT-3′; antisense 5′-ACG UGA CAC GUU CGG AGA ATT-3′) purchased from GenePharma (Shanghai, China). Transfections were performed using jetPRIME reagent (114-15, Polyplus Transfection, France) in accordance with the manufacturer’s instructions (The knockdown efficiency of SiTREM2 has been verified). Myelin debris was prepared from whole brains of mixed-sex C57BL/6 mice aged 6-8 weeks as reported previously [7]. Brain tissue was homogenized on ice in 0.32 M sucrose solution and subjected to sucrose density gradient centrifugation following established procedures. Myelin-enriched fractions were collected, washed thoroughly with cold PBS, and pelleted by centrifugation. Purified myelin fractions were resuspended in PBS and incubated with 3,3′-Dioctadecyloxacarbocyanine perchlorate (DIO; C1993S, Beyotime, China) at 37 °C for 20 min in the dark, ensuring uniform lipid incorporation [8]. Excess dye was removed by repeated centrifugation and washing with PBS until the supernatant became clear. For all in vitro assays, DIO-labeled myelin debris was added to microglial cultures at a final concentration of 1 mg/mL, 24 hours after siRNA transfection [8,9]. Western blotting was performed 24 hours post-myelin treatment, as previously detailed [9], to verify the efficiency of SiRNA knockdown. Cells were collected at 0, 3, or 5 days post-myelin treatment for further analysis, as previously detailed [8]. To assess microglial phagocytic capacity, ten Phalloidin⁺ cells were randomly selected in each sample, and the fluorescence intensity of DiO-labeled myelin within these cells was quantified. Three independent in vitro cultures were included in the experiment, and all measurements were performed in a blinded manner.

**Reference**

1. Schafer DP, Lehrman EK, Heller CT, Stevens B. An Engulfment Assay: A Protocol to Assess Interactions Between CNS Phagocytes and Neurons. *J Vis Exp*. 2014;(88):51482.

2. Love MI, Huber W, Anders S. Moderated estimation of fold change and dispersion for RNA-seq data with DESeq2. *Genome Biol*. 2014;15(12):550.

3. Benjamini Y, Drai D, Elmer G, Kafkafi N, Golani I. Controlling the false discovery rate in behavior genetics research. *Behav Brain Res*. 2001;125(1-2):279-284.

4. Draghici S, Khatri P, Tarca AL, et al. A systems biology approach for pathway level analysis. *Genome Res*. 2007;17(10):1537-1545.

5. Ashburner M, Ball CA, Blake JA, et al. Gene Ontology: tool for the unification of biology. *Nat Genet*. 2000;25(1):25-29.

6. Du S, Xiong S, Du X, Yuan TF, Peng B, Rao Y. Primary Microglia Isolation from Postnatal Mouse Brains. *J Vis Exp*. 2021;(168):62237.

7. Yao F, Luo Y, Liu YC, et al. Imatinib inhibits pericyte-fibroblast transition and inflammation and promotes axon regeneration by blocking the PDGF-BB/PDGFRβ pathway in spinal cord injury. *Inflamm Regen*. 2022;42(1):44.

8. Liu Y, Yao F, Li Z, et al. Dynamic phosphorylation of Fascin-1 orchestrates microglial phagocytosis and neurological recovery after spinal cord injury. *J Neuroinflammation*. 2025;22(1):121.

9. Yu S, Cheng L, Tian D, et al. Fascin-1 is Highly Expressed Specifically in Microglia After Spinal Cord Injury and Regulates Microglial Migration. *Front Pharmacol*. 2021;12:729524.
